# Revisiting the chlorophyll biosynthesis pathway using genome scale metabolic model of *Oryza sativa japonica*

**DOI:** 10.1038/srep14975

**Published:** 2015-10-07

**Authors:** Ankita Chatterjee, Sudip Kundu

**Affiliations:** 1 ^1^Department of Biophysics, Molecular Biology and Bioinformatics, University of Calcutta India; 2 Center of Excellence in Systems Biology and Biomedical Engineering, TEQIP Phase-II, University of Calcutta India

## Abstract

Chlorophyll is one of the most important pigments present in green plants and rice is one of the major food crops consumed worldwide. We curated the existing genome scale metabolic model (GSM) of rice leaf by incorporating new compartment, reactions and transporters. We used this modified GSM to elucidate how the chlorophyll is synthesized in a leaf through a series of bio-chemical reactions spanned over different organelles using inorganic macronutrients and light energy. We predicted the essential reactions and the associated genes of chlorophyll synthesis and validated against the existing experimental evidences. Further, ammonia is known to be the preferred source of nitrogen in rice paddy fields. The ammonia entering into the plant is assimilated in the root and leaf. The focus of the present work is centered on rice leaf metabolism. We studied the relative importance of ammonia transporters through the chloroplast and the cytosol and their interlink with other intracellular transporters. Ammonia assimilation in the leaves takes place by the enzyme glutamine synthetase (GS) which is present in the cytosol (GS1) and chloroplast (GS2). Our results provided possible explanation why GS2 mutants show normal growth under minimum photorespiration and appear chlorotic when exposed to air.

Chlorophyll, one of the tetrapyrroles synthesized in green plants, is the major light harvesting pigment^1^. It traps and converts sunlight into chemical energy that is directly or indirectly used by all other living organisms on earth. Thus, understanding the mechanism of chlorophyll biosynthesis has been a topic of research worldwide.

The biochemical consequences of chlorophyll synthesis should be linked with the central carbon metabolism and other metabolic processes spanned over different cellular compartments, in terms of photosynthesis. Therefore, the involvement of cellular metabolism in chlorophyll biosynthesis requires exploration. However, this knowledge is quite limited as the focus of previous research works were mainly centered on the chlorophyll biosynthesis pathway starting from glutamate (Glu) within the chloroplast. Several groups have studied the enzymes involved in the chlorophyll biosynthesis pathway and their regulations^2^,^3^. Three plastid compartments namely the thylakoid, stroma and the envelope membrane are known to take part in the process of tetrapyrrole synthesis^4^,^5^. The conventional pathway of tetrapyrrole biosynthesis in plants^6^ can be found as Supplementary Fig. S1 online. As shown in Fig. S1 (step 13), conversion of Mg protoporphyrinIX (MgPP) to Mg-protoporphyrin-monomethyl-ester (MgPPME) also involves conversion of S-adenosylmethionine (SAM) to adenosylhomocysteine (AdoHcy). Thus, SAM is one of the essential intermediates for chlorophyll biosynthesis pathway, which indicates that there must be a link between the synthesis of SAM in the cytosol and the chlorophyll synthesis in the chloroplast. This is discussed in the later section. The final step in synthesis of chlorophyll involves esterification of chlorophyllide a (Chld a) with phytyl pyrophosphate^7^ (PhyPP). This reaction is catalyzed by chlorophyll synthase^7^. PhyPP is derived from isoprenoid pathway. Two pathways for isoprenoid synthesis exists in plants – the mevalonate (MVA) pathway, which is localized in the cytosol and the 2-C-methyl-D-erythritol-4-phosphate (MEP) pathway/1-deoxy-D-xylulose-5-phosphate (DXP) pathway, which is localized in the plastids of a plant cell^8^,^9^. When we trace back the chlorophyll synthesis pathway we see that 5-aminolevulinic acid (ALA) is formed from Glu. This conversion of Glu to ALA is well-documented in literature; however, the source of chloroplastic Glu is not explicitly mentioned in the existing literature to describe the complete chlorophyll biosynthesis pathway and also how much of the central carbon metabolism from other compartments are involved in it, is also not studied in detail. Therefore, we curated the existing genome scale metabolic model of rice leaf^10^ and consequently studied the complete chlorophyll biosynthesis pathway. Our analysis established how the inorganic nutrients are utilized through a series of biochemical reactions localized in different cellular compartments to synthesize chlorophyll. Our study also revealed the enzyme-coding genes essential for chlorophyll biosynthesis and we further provided some experimental observations in support of the validation of their essentiality. The genes whose associated reactions, if blocked, result in no chlorophyll synthesis are termed here as ‘essential’. We also studied the activity of some intracellular transporters in chlorophyll synthesis pathway by varying the flux through the cytosolic and chloroplastic ammonia transporters. Finally, the activity of cytosolic and chloroplastic glutamine synthetase (GS1 and GS2 respectively) was analysed. By varying the source of ammonia transporters we studied the effect of GS1 and GS2 on chlorophyll synthesis pathway.

It is to mention that we considered only the chlorophyll a synthesis pathway for the present study. Chlorophyll b, which also occurs in plants, is synthesized from chlorophyll a through a series of reactions commonly referred to as *chlorophyll cycle*^11^. However, we did not include the chlorophyll cycle in our study.

Genome scale metabolic models (GSMs) represent a list of all the reactions and the associated metabolites which are typically between hundreds to thousands. Availability of complete genomes of different species and annotation of genes (gene-protein-reaction) encourage several researchers to construct GSMs. The first ever reported GSM to be published was of *Haemophilus influenza*^12^. So far, GSMs of several species have been reported including *E.coli*^13^, other bacterias^14^,^15^,^16^,^17^, *Arabidopsis thaliana*^18^,^19^, rapeseed^20^, rice^10^ and for leaf, embryo and endosperm of maize^21^. In addition, the whole plant scale metabolic model of barley (*Hordeum vulgare*) helps to understand the metabolic behavior of source and sink organs during its generative phase^22^.

There is also a constant effort to further curate the previously reported GSMs and to analyze them to address new scientific questions. For example, the Arabidopsis model (AraGEM)^23^ was revised to construct a C_4_ genome scale model (C4GEM)^24^. A more comprehensive GSM for maize was reported in 2011^25^. Recently, a further curated maize leaf model was developed^26^ by incorporating more compartments to understand the nitrogen metabolism.

Flux balance analysis (FBA) has been extensively used^27^ to model organism specific metabolism and to simulate the internal flow of metabolites. The method is based on an assumption that the production and consumption of all internal metabolites is stoichiometrically balanced and the reactions are thermodynamically feasible. It utilizes linear programming (LP) to optimize an objective function as nutrients are converted into biomass and excretory products. Different objective functions are used by different groups. While maximization of the biomass yield using the nutrients is a commonly used objective function^28^,^29^, many groups use minimization of the total flux as their objective function^10^,^18^,^30^.

## Results and Discussion

### Extending and updating the rice genome scale metabolic model

We curated the existing GSM of *Oryza sativa japonica*^10^ to include chlorophyll synthesis pathway reactions within the chloroplast module, a few transporters, and peroxisome compartment with associated reactions of photorespiration and deletion of non-plant reactions. The current model consists of 1721 reactions and 1544 metabolites. The model is capable of producing all the necessary biomass components including chlorophyll. 55 non-plant reactions were deleted (Source: BRENDA^31^) from the previous model (Supplementary Table S1 online). It is worth mentioning that there may still exist some other non-plant reactions in our model. However, our predicted chlorophyll synthesis pathway does not contain any non-plant reactions. Table 1 represents the main differences between the previous and the new model.

### Possible Reaction Set for Chlorophyll Biosynthesis

**Figure 1:**
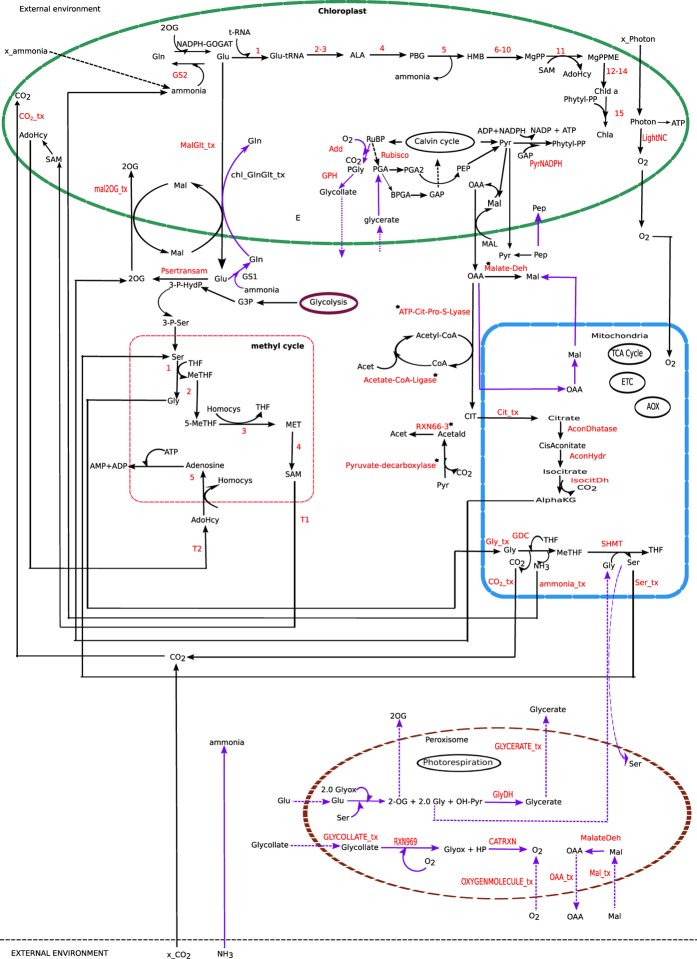
One of the possible pathways for chlorophyll biosynthesis in plants. Reactions marked in red are the essential reactions (including transporters). For reactions numbered 1–15 refer Supplementary Figure S1 online. The three compartments, i.e. chloroplast, mitochondria and the cytosol were found to be involved in chlorophyll synthesis pathway. When Rubisco oxygenase activity comes into play, the peroxisome is also involved in the process, wherein a few of the photorespiratory reactions are also found to be essential. Bold purple arrow heads indicate the reactions involved when Rubisco carboxylase and oxygenase was set at 3:1, broken purple arrows indicate peroxisomal transporters. Methyl cycle is found to be essentially linked with chlorophyll synthesis. *Intermediates*: 3-P-HydP, 3-P-HYDROXYPYRUVATE; 3-P-Ser, 3-PHOSPHO-SERINE; HP, Hydrogen-peroxide; OH-Pyr, Hydroxy-pyruvate; 2OG, oxoglutarate; SAM, S-AdenosylMethionine; AdoHcy, AdenosylHomoCysteine; Hcy, homocysteine; Gly, glycine; AlphaKG , Alpha-ketoglutarate/oxoglutarate; Ser, serine; PEP, phosphoenol pyruvate; OAA, oxaloacetate; Mal, malate; Glu, glutamate; Gln, glutamine; Pyr,pyruvate; THF,tetrahydrofolate; MeTHF,5,10-methylenetetrahydrofolate; 5-MeTHF,5-methyltetrahydrofolate; MET,methionine. *Enzymes of methyl cycle*: 1, 5,10-methylenetetrahydrofolate:glycine hydroxymethyltransferase; 2, 5,10-methylenetetrahydrofolate reductase; 3, methionine synthase; 4, S-adenosylmethionine synthetase; 5, adenosylhomocysteinase. Enzymes: GS2, chloroplastic glutamine synthetase; GS1, cytosolic glutamine synthetase. *External metabolites*: x_CO2- external carbon-dioxide; x_Ammonia- external ammonia. _tx indicate the transporters. Reactions marked *are the reactions found to be essential but no supporting literature is available.

Using FBA we identified one of the possible pathways for chlorophyll synthesis (Fig. 1).

This pathway has 108 reactions among which 22 are cytosolic, 57 are chloroplastic, 10 are mitochondrial and 19 are intra cellular transporters (Supplementary Table S2 (a) online). Here, we did not couple the RuBP carboxylase and oxygenase reactions in any fixed ratio; rather they were used as two independent reactions. The objective function used was the minimization of the total flux which represents the economy of the enzymic machinery^10^.

The photon flux needed to produce one unit of chlorophyll is nearly 666.5 light flux unit and the flux values through light non-cyclic and cyclic reaction were 44.42 and 3.17 unit, respectively.

### Elucidating the complete biosynthesis pathway of chlorophyll

We already mentioned that the previous works mainly focused on the chlorophyll synthesis pathway starting from chloroplastic Glu, which is a precursor of chlorophyll biosynthesis. Here, briefly we describe that the chlorophyll biosynthesis pathway is not limited to the chloroplastic compartment, rather it is distributed in different other sub-cellular compartments namely the chloroplast, mitochondria and cytosol. Detail description can be found as Supplementary Discussion S1 online.

8.0 unit of Glu in the chloroplast is needed for chlorophyll synthesis. The source of this chloroplastic Glu can be either ferredoxin dependent GOGAT (EC 1.4.7.1) or NADPH dependent GOGAT (EC 1.4.1.13). Our result showed the involvement of the NADPH dependent GOGAT (Fig. 1). This GOGAT was linked with the chloroplastic Mal-Glu and Mal-2OG transporters. In the chloroplast, one of the important steps of chlorophyll biosynthesis is the conversion of MgPP to MgPPME, which is associated with SAM to AdoHcy conversion (Fig. 1). Consequently, the pathway showed the involvement of the transporters for SAM and AdoHcy and the methyl cycle in the cytosol. Since plant chloroplast and mitochondria lack AdoHcy hydrolase^32^, the transport of AdoHcy from the chloroplast to the cytosol is required for methyl cycle to operate^32^. Further, it has been reported that impairment of SAM transporter affects plastid biogenesis^33^ and moreover, lack of SAM synthetase in the chloroplast^32^ implies the import of SAM into the chloroplast from the cytosol. OAA is imported into the cytosol via the chloroplastic Mal-OAA shuttle. OAA in the cytosol participates in giving Mal and CIT. The CIT is transported into the mitochondrion and consequently forms 2OG, which is transported out of the mitochondrion via a transporter (Fig. 1).

The result also showed involvement of Ser-Gly conversion in the cytosol, which is obligatory for one carbon metabolism^34^. Gly from the cytosol enters into the mitochondria, where it participates in GDC (EC 1.4.4.2) and SHMT (EC 2.1.2.1) mediated reactions. Ser from the mitochondria comes out into the cytosol and the mitochondrial ammonia enters into the chloroplast to participate in Gln synthesis.

### Essential Reactions, associated genes and comparison with experimental observations

The in-silico reaction deletion strategy was used to identify the essential set of reactions for chlorophyll biosynthesis. We first identified the essential reactions when RuBP carboxylase and oxygenase activity were represented as two independent reactions. A total of 85 reactions were identified as essential, distributed in all compartments. Among them 46 are chloroplastic, 5 are mitochondrial and 17 are cytosolic reactions. A total of 17 intracellular transporters were identified as essential. We also identified that a total of 148 genes are associated with these essential reactions. A list of all essential reactions and associated genes is given in Supplementary Table S2 (b) online. When we combined the RuBP carboxylase and oxygenase reactions and simulated the metabolism, the results showed a few more essential reactions specific to peroxisome (and a few chloroplastic), primarily because of the involvement of photorespiration (see Supplementary Table S2 (c) online). The effect of photorespiration is discussed later.

Here, we report experimental evidences supporting a relationship between the decreased expression (or no expression) of a large number of essential genes with reduced chlorophyll content in plant leaves (Table 2). Some of the genes, when mutated or knocked out, caused reduced chlorophyll content. On the other hand, several experiments have demonstrated a sharp decline in chlorophyll content (known as chlorosis) under stress as well as decreased expressions of some of the essential enzymes. It is to mention that one of the major causes of chlorosis is a very fast breakdown of chlorophyll and this is associated with alteration in the overall metabolism, the effect of which is discussed later.

Among the predicted essential genes, the participation of 20 chloroplastic enzymes in chlorophyll synthesis has been reported by several groups^3^,^4^,^8^,^35^,^36^ (Supplementary Table S3 online). One would further expect the transport of CO_2_, O_2_ and light (light transporter has been added for modeling purpose) into the cell to be essential for the cellular metabolism. In addition, we found chloroplastic transporters for SAM, AdoHcy, Mal-Glu and Mal-2OG also essential along with some reactions of Calvin cycle (glyceraldehyde-3-phosphate dehydrogenase, transketolase, sedoheptulose-bisphosphatase, phosphoribulokinase, phosphoglycerate kinase and Ribulose-bisphosphate carboxylase).

We found a total of 12 mitochondrial reactions (7 intra-cellular transporters and 5 reactions within mitochondria) to be essential. These mitochondrial reactions are catalyzed by 4 different enzymes - aconitate hydratase (AH), glycinedecarboxylase (GDC), 5,10 methylenetetrahydrofolate:glycinehydroxymethyltransferase/serine hydroxymethyltransferase (SHMT) and isocitrate dehydrogenase (ICDH). These results are in accordance with a few experimental studies those have reported that if the plants are deficient in these enzymes, they would have less or no chlorophyll (i.e. chlorosis), depending on the amount of enzyme available for carrying the reaction; hence, they would appear chlorotic^37^,^38^,^39^. Mutants with reduced level of T-protein subunit of GDC were also reported to show chlorosis^35^. Further, the reduced expression of the enzyme ICDH, localized within plant mitochondria and involved in TCA cycle, was also reported to cause decrease in chlorophyll a and b content^38^. Mutation in SHMT was also reported to cause chlorosis^39^.

In the cytosol, as described in the previous section, we found the methyl cycle to be active. Additionally, we found the enzymes associated with methyl cycle to be essentially linked with chlorophyll synthesis. This is because methyl cycle is required for the synthesis of SAM in the cytosol, which is transported into the chloroplast for the methylation step for chlorophyll synthesis (EC 2.1.1.11). Thus we can infer a direct link between methyl cycle and chlorophyll synthesis. This result is in accordance with the study that had reported the effect of THF synthesis inhibition on chlorophyll production in pea plant^40^. They observed a considerable amount of decrease in the rate of chlorophyll synthesis, and demonstrated how reduced concentration of 5-MethylTHF affects the methyl cycle and consequently chlorophyll synthesis.

Reduced expression of chloroplastic geranyl reducatse (CHL P) was studied by expressing antisense CHL P RNA and it was reported that chlorophyll content in the transformants decreased^36^. Dalal and Tripathy had reported a sharp decline in chlorophyll content under water stress^41^. A substantial decrement was also observed in the activity of the enzymes – ALA-Dehydratase, PBGD/HMBS, COPRO, Porphyrinogen IX oxidase, Mg chelatase, geranylgeranyl reductase and POR under water stress. They argued that a sharp decline in chlorophyll content was possibly due to the decreased accumulation of certain chlorophyll synthesis intermediates. Understandably, since the flux through any enzymatic reaction depends on several factors including the enzyme’s concentration, the lower enzymatic concentration of the protein might reduce the flux through the respective reaction. However, we should mention that although reduced chlorophyll synthesis might be one of the causes of chlorosis, several studies reported that plants subjected to different stresses including metal toxicity, osmotic stress etc. show chlorotic leaves due to enhanced chlorophyll degradation^42^,^43^,^44^. Moreover, some proteins degrade at a very fast rate under stress. The protein degradation causes generation of reactive oxygen species (ROS) which must have an overall impact on cellular metabolism under stress conditions. Here, neither the chlorophyll breakdown nor the possible impact of ROS and nitrogen species was considered. In fact, one might expect a complicated interaction of the cellular metabolism under stress. Finally, we want to point out that a large number of essential genes, predicted in our analysis, have been reported to show reduced expression while the phenotypic changes observed in leaves were of low chlorophyll content. Moreover, although there is no linear relationship between gene expression and enzyme kinetics of enzymatic genes, yet one might expect that lower enzymatic concentration might influence the flux through the respective reaction. Thus, the set of essential genes that we predicted would provide a new insight in understanding the chlorophyll biosynthesis pathway in plants. When we considered both the Rubisco carboxylase and oxygenase activities, we additionally identified a set of peroxisomal reactions (including 6 transporters) as essential. The basis of this additional essentiality is that the cellular metabolism is bound to complete the C2 cycle^44^.

### When Rubisco has both carboxylase and oxygenase activity

Rubisco has both carboxylase and oxygenase activity and its ratio (Vc/Vo) at normal condition is three^45^. Although we considered both the reactions representing Rubisco carboxylase and oxygenase activity, but, since they were not coupled, and the oxygenase reaction and the associate C2 cycle (photorespiration) cause loss of CO_2_ and NH_3_ in intermediate steps of C2 cycle, the simulation always preferred to include only Rubisco carboxylase activity. Here, we fixed the carboxylation to oxygenation flux ratio (Vc/Vo) of Rubisco at 3:1.

The results showed involvement of 128 reactions in one of the possible pathways for chlorophyll synthesis. 99 reactions were found to be essential which spanned over the chloroplast, cytosol, mitochondria and peroxisome. Figure 1 describes the chlorophyll synthesis pathway when Rubisco carboxylase/oxygenase activity was set at ratio 3:1. As expected, due to Rubisco oxygenase activity the essential reactions included 6 peroxisomal transporters, 5 peroxisomal reactions, chloroplastic transporter for glycollate to peroxisome, conversion of glycerate to PGA (EC 2.7.1.31) in the chloroplast and conversion of phosphoglycollate to glycollate in the chloroplast (EC 3.1.3.18) (Supplementary Table S2 (c) online).

The photon flux needed was 1092 light flux unit, which was much higher than what we got with only carboxylase activity, i.e. 666.5 light flux unit. The increase in the photon flux was expected due to the involvement of photorespiration. The cytosolic ammonia transporter instead of the chloroplastic ammonia transporter was active along with the chloroplastic Glu-Gln shuttle.

### Allowable range of flux values of each reaction of the solution space

We performed flux variability analysis (FVA) that calculated the allowable range of flux values that a reaction can carry while achieving the optimal objective value. In our analysis, the minimization of the total flux values of the reactions was set as the primary objective and the flux through the chlorophyll transporter was set to one unit. We observed no variability in the minimum (FVA_min_) and maximum (FVA _max_) flux values of the reactions. Rather, both the FVA_min_ and FVA _max_ values of all the reactions were equal to the flux values that were obtained from FBA analysis (FBA_val_) (Supplementary Data S1 online). However, a plant cell might not always try to optimize its cellular economy which has been represented as a minimization of the total cellular flux values. To address this issue, the primary objective function was set at 1.5 and 2.0 times to the computed optimal value (that was obtained under minimization of the total cellular flux), separately and simulated the cellular metabolism. We observed two kinds of results:

(i) For some of the reactions such as chl_FPPSYNRXN, chl_PROTOPORGENOXIRXN etc. FVA_min_ = FVA_max_ = FBA_val_, while for others such as mit_AconDHatase, mit_AconHydr etc. FVA_min_ = FBA_val_ < FVA_max_. Both the cases indicate that these reactions are essential for chlorophyll biosynthesis. The same set of essential reactions was predicted in reaction deletion study (discussed earlier).

(ii) For the remaining reactions we observed that FVA_min_ < FBA_val_ < FVA_max_. A set of reactions (irreversible) had FVA_min_ = 0, while the set of reversible reactions had FVA_min_ < 0, indicating the later can carry flux in both the directions. Both the sets of reactions indicate that these are not essential reactions for chlorophyll synthesis. The reactions carrying variable flux values form alternate metabolic routes and thus indicate the presence of metabolic redundancy in the system^46^.

Interestingly, we observed that the external ammonia transporter had FVA_min_ and FVA_max_ equal to 0 and 4 flux unit, respectively. As the flux value of the chlorophyll transporter was set to 1.0 unit in our analysis, the external ammonia that should be transported through the transporters to the cell should be 4.0 flux unit. This ammonia can be transported through either the chloroplastic or the cytosolic ammonia transporter or both. We also observed variations in the fluxes of some of the intracellular transporters.

### Activity of intracellular transporters and their association with ammonia transporters

Nitrogen is one of the essential macronutrients and plays a major role in plant growth and crop productivity. Plants take up nitrogen from the soil in two inorganic forms, ammonium ion (NH_4_^+^) and nitrate ion (NO_3_^−^). Rice is cultivated in flooded paddy fields. The fact that it prefers ammonium as the nitrogen source is well established^47^. Ammonium also constitutes the major form of nitrogen in the bulk soil^48^. Another reason behind ammonia being preferred over nitrate is the lower energy requirement for its assimilation^49^. Use of ammonia based fertilizers is also in practice to achieve high yield rice variety^50^. Ammonia assimilation thus plays an important role in rice plant growth and productivity.

The ammonia assimilation occurs in the roots, leaves and shoots of plants. It is taken up by plant cells via ammonium transporters and distributed to different intracellular compartments including chloroplast and cytosol. Most of the ammonia transported into a plant cell is assimilated via glutamine synthetase^51^ (GS; EC 6.3.1.2) forming glutamine (Gln), which thereafter in combination with 2OG forms Glu and the reaction is catalyzed by glutamate synthase (GOGAT). These two reactions form a cycle, referred as the GS/GOGAT pathway^52^. The focus of our present work is centered on rice leaf metabolism. Two GS isoenzymes are present in rice leaves, one in the chloroplast (GS2) and a second in the cytosol^53^ (GS1). In fact, GS2 is found to be rare in rice root but is abundant in rice leaves^54^ .The relative amounts of these isoenzymes change with leaf development^55^. Moreover, it is also reported that there are different isoforms of GS1^56^ and their activities depend on the cellular conditions. For the sake of simplicity, we have only considered the presence of two isoforms of GS—one in the chloroplast and another in the cytosol. While GS1 is important for plant’s normal growth and development^57^, GS2 is needed for photorespiratory metabolism of nitrogen in chloroplast^58^.

We considered two transporters for external ammonia – (i) into the cytosol (NH_3__tx) (ii) into the chloroplast (ex_ammonia_tx). First, we aimed at obtaining a solution space without fixing the flux through both the ammonia transporters.

The FBA showed that while the transport of ammonia was equally possible through both the transporters, the cytosolic ammonia transporter was preferred over the chloroplastic ammonia transporter when both the Rubisco’s carboxylase and oxygenase activities were considered. On the other hand, when we considered only the Rubisco’s carboxylase activity, the chloroplastic ammonia was preferred. We want to mention here that at normal air, the Rubisco enzyme has both the carboxylase and oxygenic activities. The ammonia in the cytosol participates in Gln synthesis (GS1) (Fig. 1). This indicates a possible link between GS1 in the cytosol and chlorophyll synthesis pathway. Very recently, a similar study showed that the cytosolic GS mutant causes lower biosynthesis of chlorophyll in *Zea mays*^26^.

Further, in order to study the effect of relative variation in the use of ammonia transporters (and hence of GS activity), we simulated the cellular metabolism by varying the flux through the chloroplastic ammonia transporter from 0 to 4 under two different conditions: case 1: without Rubisco oxygenase activity and case 2: with Rubisco oxygenase activity. The range of flux (0 to 4) through ammonia transporter reflects the constraint used in our simulation, i.e. we simulated the metabolism to synthesize one unit of chlorophyll, which consists of four nitrogen atoms.

For case 1, the flux through GOGAT (EC 1.4.1.14) was found to be fixed at 8.5 throughout the simulation. The methyl cycle in the cytosol was found to be linked with the mitochondrial GDC and SHMT via the mitochondrial Gly and Ser transporters. The results showed that the mitochondrial GDC and SHMT had a flux of 0.5 unit, and consequently, the ammonia was released in the mitochondria (via GDC). This ammonia of 0.5 unit flux transports into the chloroplast.

**Figure 2:**
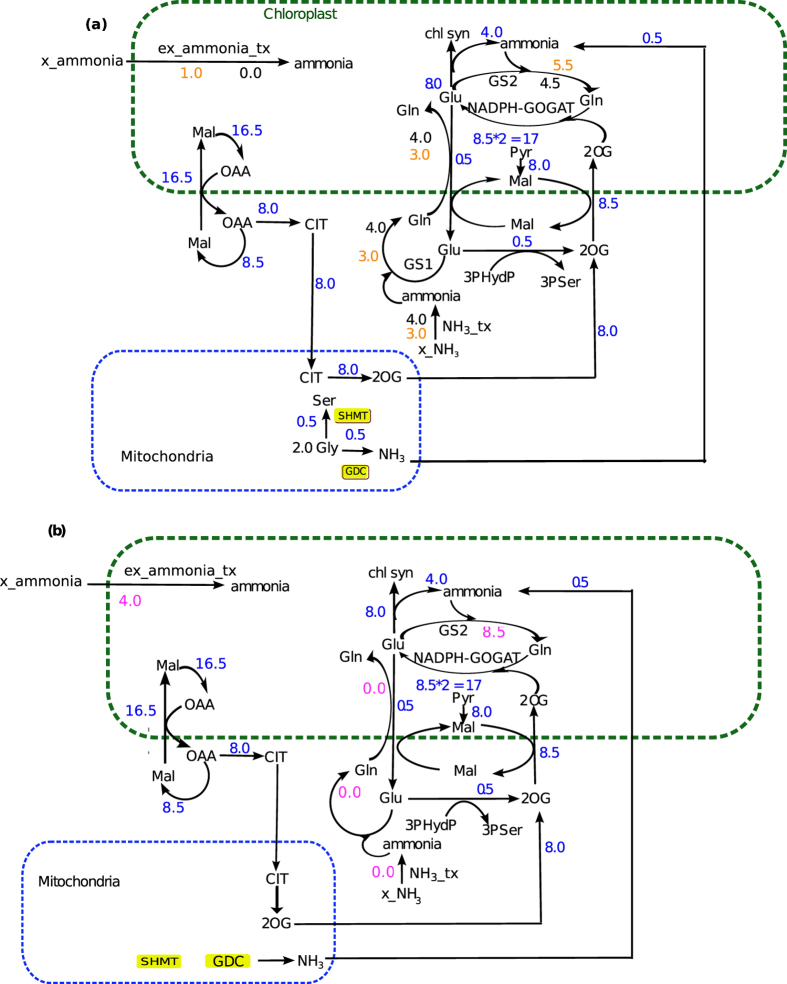
Activity of various intracellular transporters with only Rubisco carboxylase activity. (**a**) Direct interlink was found between GOGAT and Mal-2OG transporter. 8.5 unit of 2OG always entered into the chloroplast by the Mal2OG transporter and same unit of 2OG is consumed in GOGAT. The flux through GOGAT is also 8.5 throughout. Flux values marked in blue are the ones that were found to be same throughout the simulation under Rubisco carboxylase activity. When the flux through the chloroplastic ammonia transporter (ex_ammonia_tx) was set to zero (flux values marked in black under this condition) then 4.0 unit of flux passed through the cytosolic ammonia transporter (NH_3__tx), chloroplastic Glu-Gln shuttle and the cytosolic GS. Further, when the flux through the chloroplastic ammonia transporter was increased by 1.0 unit (flux values marked in orange under this condition) consequently the flux through cytosolic ammonia transporter, chloroplastic Glu-Gln shuttle and the cytosolic GS decreased by 1.0 unit. The increase in the chloroplastic ammonia by 1.0 unit also increased the flux through the GS2 by 1.0 unit. The results indicated that the activity of chloroplastic Glu-Gln transporter and source of chloroplastic ammonia was coupled. (**b**) As the activity of the chlroplastic ammonia transporter was increased the flux through the NH_3__tx, cytosolic GS and chloroplastic Glu-Gln shuttle decreased. Thus, when chloroplastic ammonia transporter was fixed at 4.0 unit (flux values marked in pink under this condition) the NH_3__tx, Glu-Gln transporter and cytosolic GS carried zero flux. The total chloroplastic ammonia in this case was 8.5 unit and was consumed in the GS2. *Metabolites*. Gln, glutamine; Glu, glutamate; Mal, malate; OAA, oxaloacetate; CIT, citrate; 2OG, Oxoglutarate, Gly, glycine; Ser, serine; Pyr, pyruvate. *Enzymes*. GDC, glycine decarboxylase; SHMT, serine hydroxylmethyltransferase; MDH, malate dehydrogenase; GOX, glycolate oxidase; CAT, catalase.

Next, two different physiological conditions were imposed on the model to obtain the solution space—(1) activity of cytosolic ammonia transporter was fixed at 4.0, while chloroplastic ammonia transporter was blocked and (2) vice-versa—and the corresponding results were analyzed. The two conditions showed the maximum effect on the activities of the chloroplastic Glu-Gln transporter, GS1 and GS2. The flux through chloroplastic Glu-Gln shuttle was maximum while the flux through cytosolic ammonia transporter was fixed at 4.0 unit and the former gradually decreased with the reduction of the later (Fig. 2a). The flux through GS2, on the other hand, was minimum when the flux through cytosolic ammonia transporter was fixed at 4.0 unit (Fig. 2a) and the former gradually increased by 1.0 unit with decrease in the flux through the later and a simultaneous increase in the flux through the chloroplastic ammonia transporter (Fig. 2a). When the flux through the chloroplastic transporter was fixed at 4.0 unit (second condition), we found that the chloroplastic Glu-Gln shuttle became inactive (Fig. 2b). GS2 carried a flux of 8.5 unit, equal to the flux through GOGAT pathway (Fig. 2b). It is very much interesting to note that the activity of the chloroplastic Glu-Gln depends on the activity of GS1; when the flux through the later is 0, the former becomes completely inactive.

**Figure 3:**
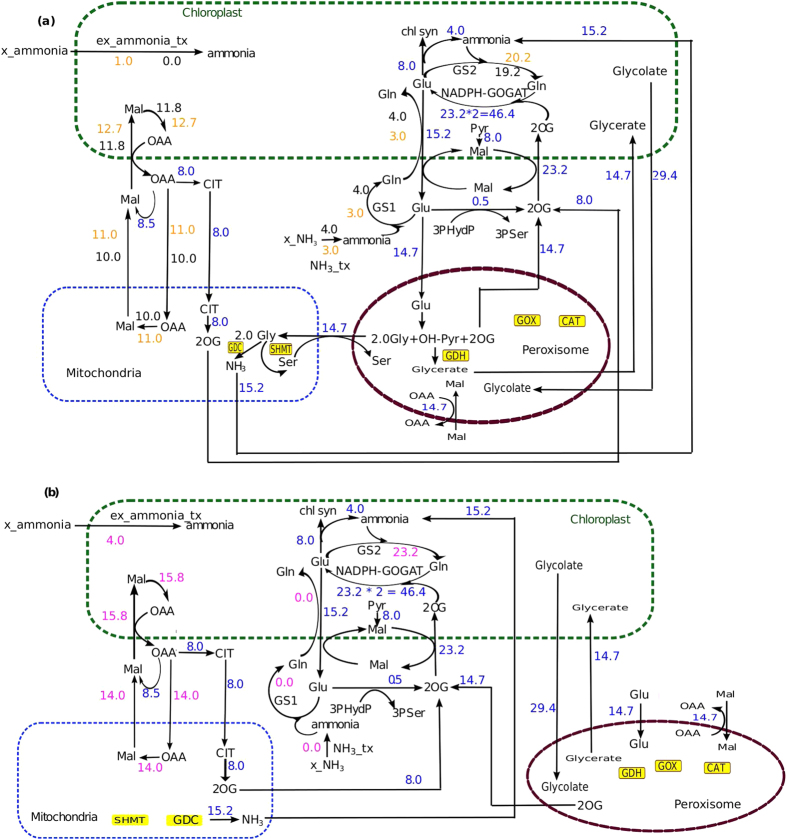
Activity of various intracellular transporters under combined activity of Rubisco carboxylase and oxygenase. Flux values marked in blue are the ones that were found to be same throughout the simulation under combined activity of Rubisco carboxylase and oxygenase. Similar to what we found under Rubisco carboxylase activity, same interlink was observed between chloroplastic GS and Mal-2OG transporter; chloroplastic ammonia transporter (x_am_tx) and chloroplastic Glu-Gln (the flux values increased due to photorespiration). In addition, the reactions of the peroxisome also were involved. (**a**) Fluxes through various transporters when flux through the chloroplastic ammonia transporter (ex_ammonia_tx) was set at 0 and 1 flux unit. (**b**) Fluxes through various transporters when flux through the chloroplastic ammonia transporter (ex_ammonia_tx) was set at 4 flux unit.

For case 2, i.e. in the presence of Rubisco oxygenase activity, as expected to complete the C2 cycle, the fluxes through the chloroplastic transporter for Mal-Glu and Mal-2OG and the mitochondrial ammonia transporter, SHMT and GDC were much higher compared to what we obtained in case 1 (Fig. 3a).

While we varied the fluxes through cytosolic and chloroplastic ammonia transporters in Case 2, we observed the similar trend (as of Case 1) (Fig. 3a,b).

For the GOGAT catalyzed reaction (EC 1.4.1.14) 2OG is required as a substrate. Therefore, the import of 2OG into the chloroplast had to be equal to the flux of GOGAT reaction. Thus, in case 1, the flux through the chloroplastic Mal-2OG was always 8.5 unit and in case 2 (involving photorespiration) it was 23.2 unit.

Thus, on the basis of the results we can infer that under the relative activity of the ammonia transporters, there exists a clear and an important interlink between the various intracellular transporters (Glu-Gln, Mal-Glu and Mal-2OG), in order to complete the chlorophyll biosynthesis pathway. In specific, while the chloroplastic Mal-Glu and Mal-2OG transporters are always active and essential for chlorophyll biosynthesis, the activity of chloroplastic Glu-Gln depends on the relative activities of GS1 and GS2.

### Chloroplastic glutamine synthetase (GS2) and chlorophyll synthesis

It is known that GS2 is needed for photorespiratory metabolism of nitrogen in chloroplast^58^. A study on barley GS2 mutant reported that plants grew normally under high CO_2_ condition (i.e. minimum photorespiration) but appeared chlorotic when exposed to air^58^. The study confirmed ammonia accumulation in the barley leaves when exposed to air^58^.

**Figure 4:**
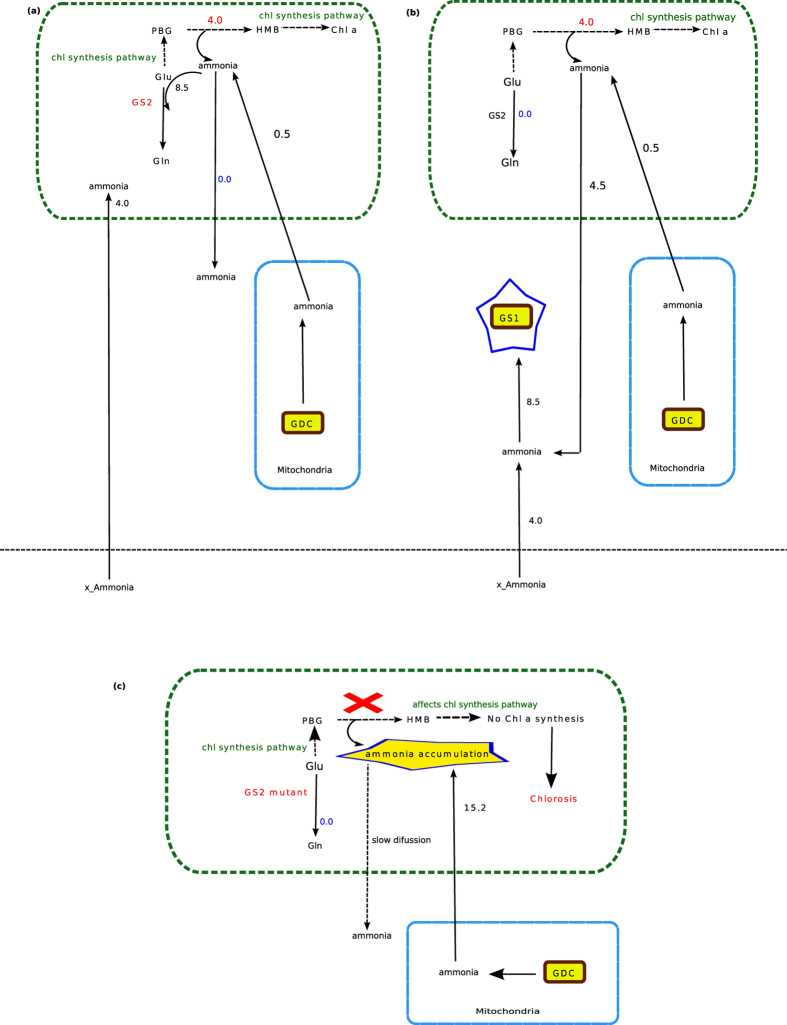
Activity of GS2 and chlorophyll synthesis. (**a**) When the transport of chloroplastic ammonia to the cytosol was not considered, then the activity of GS2 was found to be essential (marked in red). Since Rubisco oxygenase activity was not involved, only 0.5 flux unit of mitochondrial ammonia was released into the chloroplast. The chloroplastic ammonia released during porphobilinogen (PBG) to hydroxymethylbilane (HMB) conversion for one unit of chlorophyll synthesis is 4.0 unit.The total chloroplastic ammonia (4.5 unit) was refixed in GS2. (**b**) If the chloroplastic ammonia was allowed to transport from chloroplast to the cytosol , then, GS2 was found to be inactive. (**c**) Under combined activity of Rubisco carboxylase/oxygenase at 3:1 (photorespiration is also considered), higher amount of ammonia is released from the mitochondria into the chloroplast. GS2 mutants are unable to fix the ammonia in the chloroplast. Due to increased concentration of ammonia within the chloroplast and its slow diffusion from chloroplast to cytosol, the chloroplastic ammonia accumulates within the chloroplast which in turn makes PBG to HMB conversion unfavourable and consequently affects chlorophyll synthesis pathway and therefore causes chlorosis. Metabolites. Gln, glutamine; Glu, glutamate; PBG, porphobilinogen; HMB, hydroxymethylbilane; chl a, chlorophyll a. Enzymes. GDC, glycine decarboxylase; GS1, glutamine synthetase 1; GS2, glutamine synthetase 2.

To synthesize one flux unit of chlorophyll, 4.0 unit of ammonia is always generated in the chloroplast (PBG to HMB conversion, Fig. 1). Moreover, mitochondrial GDC (found to be essential) also releases ammonia, which is transported into the chloroplast^59^. The total chloroplastic ammonia produced for one unit of chlorophyll synthesis is 4.5 unit (in absence of photorespiration). This chloroplastic ammonia has three possible fates– it may be (i) consumed in GS2 mediated reaction or (ii) diffused into the cytosol or (iii) accumulated in the chloroplast. When we did not consider the possibility of diffusion of the chloroplastic ammonia under steady state condition, the results showed that the chloroplastic ammonia was refixed through GS2 (the only alternative) and hence was found to be essentially active throughout the simulation (Fig. 1 and Fig. 4a). On the other hand, if chloroplastic ammonia was allowed to diffuse, then, as expected, GS2 was found to be inactive (Fig. 4b) (See the solution file as Supplementary Data S2 online). We can here infer that GS2 mutants could survive under non-photorespiratory condition only if there’s a way out for the chloroplastic ammonia to diffuse out of the chloroplast. This diffusion of chloroplastic ammonia to the cytosol results in a cellular condition where ammonia released in the chloroplast would not accumulate and the plant maintains normal growth and development. In fact, in GS2 mutants, ammonia accumulation was not observed when grown under high CO_2_ condition^58^. We hypothesise that diffusion from chloroplast to the cytosol is not very fast.

Further, when we simulated the metabolism with Rubisco carboxylase/oxygenase activity fixed at 3:1 (normal air) the ammonia released from the mitochondria to the chloroplast increased (15.2 flux unit, Fig. 3) due to photorespiration, and consequently, the total chloroplastic ammonia increased. GS2 mutant plants have been seen to appear chlorotic under increased photorespiration^58^. The essential activity of GS2 (from our results) could explain the cause behind it. Our results suggested that the probable cause behind chlorosis could be that a GS2 mutant is not able to refix ammonia within the chloroplast. Due to the increased concentration of ammonia (almost four fold than what was under high CO_2_ condition) and slow rate of diffusion, the chloroplastic ammonia could not get sufficient time to diffuse into the cytosol and thus, there would be an accumulation of chloroplastic ammonia. This ammonia accumulation can be as high as 50 times (when exposed to air for 30 min) of what is observed under non-photorespiratory condition^58^. This increased chloroplastic ammonia concentration would make the PBG to HMB conversion (Fig. 4c) unfavourable and consequently affects the chlorophyll synthesis pathway and hence the chlorophyll production.

In summary, the total ammonia produced in the chloroplast and transported from the mitochondria (due to GDC activity) under only Rubisco carboxylase activity was approximately 1/4^th^ of the total chloroplastic ammonia produced under Rubisco carboxylase/oxygenase activity at 3:1 ratio. This chloroplastic ammonia, if not refixed by GS2, should either diffuse out or accumulate in the chloroplast. Ammonia accumulation in GS2 mutants has been reported^58^, indicating slow diffusion of the ammonia through the chloroplast membrane. Our results indicate that slow diffusion of chloroplastic ammonia would cause ammonia accumulation in the chloroplast, while photorespiration occurs at normal air; this accumulation would inhibit PBG to HMB conversion in the chlorophyll synthesis pathway, which would ultimately decrease chlorophyll synthesis. Finally, if the plant is exposed to the normal air for larger time, the plant would die.

## Conclusion

In this work we updated the available rice GSM by adding some reactions, intracellular transporters and peroxisome compartments and removing non plant reactions. We used FBA to simulate the metabolism for further analysis and the results allow us to conclude the following:

1. The essential reactions and their associated genes required for chlorophyll biosynthesis span over the three plant compartments (cytosol,chloroplast and mitochondria) when Rubisco oxygenase activity (i.e. photorespiration) is not considered. Peroxisome comes into play when photorespiration is active.
2. FVA indicates variations in the fluxes through the non essential reactions and existence of alternate path for chlorophyll synthesis. It also confirms the essentiality of the reactions that we predicted by deletion study.
3. Ammonia assimilation, activity of GS1 and GS2 and other intra cellular transporters are linked with the chlorophyll biosynthesis pathway. Variation in the ammonia transporters affects the activity GS/GOGAT pathway and the Glu-Gln antiporter.
4. Analysis of the activity of GS in nitrogen assimilation provides a possible explanation on why GS2 mutants show normal growth under minimum photorespiration and appear chlorotic when exposed to air.

## Methods

### An improved GSM for rice (Oryza *sativa*)

We used the GSM of *Oryza sativa japonica*^10^ which represents a developing leaf cell of rice and was primarily derived from the annotations in the Ricecyc database^60^. This model consists of 1721 reactions and 1544 metabolites and represents a network capable of producing biomass precursors (amino acids, starch, nucleic acids, lipids, glucose, sucrose, cellulose and lignin) using CO_2_ as the sole carbon source. The inputs used in the model are – water, CO_2_, nitrogen as NH_3_ and NO_3_, phosphate, sulphate and photons. Transport reactions were added in the model by which these inorganic nutrients and photon can be transported to the plant cell. The model was used to find the realistic physiological behaviour of leaf metabolism at different light intensities. However, this study did not report anything about chlorophyll synthesis pathway. We should mention that although the previous model included some of the reactions of chlorophyll synthesis pathway but, the reactions were not compartment specific. So, for the present study the model was extended and updated by (i) adding the missing reactions for chlorophyll synthesis pathway along with the existing reactions within the chloroplast module, (ii) adding the peroxisome compartment/module with photorespiration associated reactions and (iii) deleting some non-plant reactions that were identified based on the existing knowledge.

In specific, in the chloroplast module we added- nine reactions for chlorophyll synthesis in the chloroplast (Source: METACYC^61^) 6 reactions for nitrogen assimilation, a reaction catalyzed by adenylate kinase and two photorespiration specific reactions (glycerate 3 kinase and phosphoglycolate phosphatase). Similar to the previous model^10^ two lumped reactions, one for cyclic and one for non-cyclic photophosphorylation, have been used within the chloroplast to represent the light reactions.

Further, in the mitochondria module, we added two reactions associated with photorespiration- catalyzed by serinehydroxymethyl transferase (SHMT) and glycine decarboxylase (GDC). The SHMT is localized both in the mitochondrion and the cytosol of plant cell^62^ but GDC is restricted to the mitochondrion^34^. While SHMT synthesizes serine from MethyleneTHF (5,10-methylenetetrahydrofolate) and glycine , GDC catalyzes the tetrahydrofolate-dependent catabolism of Gly to MethyleneTHF and releases NADH, CO_2_, and NH_3_^34^. Both the reactions are involved in photorespiration and folate transformation.

While incorporating the peroxisome compartment we added only 5 photorespiratory reactions^44^ (glycolate oxidase, catalase, combined reaction for serine:glyoxylate aminotransferase and hydroxypyruvate reductase, malate dehydrogenase and glycerate dehydrogenase). The model files with all the reactions are available as Supplementary Data S3 online.

Further, we also added a few transporters associated with specific compartments. In the chloroplast module, we added two transporters namely T1 & T2 for SAM and AdoHcy respectively^63^. While the former is involved in the transport of the SAM from the cytosol into the chloroplast which plays a key role in chlorophyll synthesis; the latter is involved in the transport of the AdoHcy, an intermediate formed during chlorophyll synthesis, from the chloroplast to the cytosol. Further, 6 more chloroplastic transporters^63^ and 5 mitochondrial transporters^59^,^64^ were added in the model (see Supplementary Data S3 online, chltxs.xls and mito.xls respectively). List of transporters added is given as Supplementary Table S4 online.

Further, while analysing the previous model^10^ we identified a few non-plant reactions (55 reactions) and deleted them (Supplementary Table S1 online). The present model thus consists of 1721 reactions and 1544 metabolites after curation.

### Model Analysis

After the model was curated it was then checked for stoichiometry inconsistencies. Energy and redox conservations were also checked using the method described for the previous model^10^. The final curated model was then analyzed using flux balance analysis (FBA) method^65^.

FBA aims at deriving a feasible set of steady state fluxes and optimizing the objective function subjected to constraints specified. At steady state, the rate of production of each internal metabolite in the network is equal to its rate of consumption. This state can be represented mathematically as:


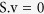


where v is a vector of fluxes through the metabolic network and S is the stoichiometry matrix^65^ (S) is an m × n matrix where m represents the rows and the corresponding metabolites and n represents the columns and the corresponding reactions.

FBA was employed both in model validation (e.g. energy and redox conservation) and analysis. We used the ScrumPy metabolic modeling package^66^ which has facilities for performing linear programming (using the Gnu Linear Programming Kit, http://www.gnu.org/software/glpk/). In our analysis, the linear programming is defined as


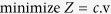



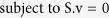



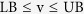


Here, Z is the objective function, v is the flux vector, c is the transpose of a vector of objective coefficients, S is the stoichiometry matrix, and LB and UB are the vectors of fluxes lower bounds and upper bounds, respectively.

In most of the analysis (unless mentioned explicitly, e.g. when we fixed the flux through the ammonia transporters) the fluxes of all nutrients and photon are not fixed. However, we have fixed the flux through the chlorophyll transporter at 1.0 unit throughout. The curated model was subjected to linear programming (LP) in order to verify whether the system represented by the model was capable of generating the biomass precursors (including chlorophyll).

To find the essential reactions for chlorophyll biosynthesis, the flux of each reaction in our predicted pathway for chlorophyll synthesis was set to zero (one at a time). Then the model was simulated taking inorganic nutrients as inputs to test whether there was a possible path for chlorophyll synthesis.

From the modeling point of view, a reaction is said to be essential if the simulation of the model does not find any possible solution space to produce the desired biomass when the flux passing through that reaction is set to zero. The genes associated with this reaction are considered as essential genes. It refers to a cellular situation where the enzymatic activities of these genes are inhibited in such a way that the reaction does not occur and the cell is not able to produce the desired biomass. On the other hand, non essential reactions are those for which the simulation finds alternative reactions to produce the desired biomass. Thus, while the essentiality refers to the non-existence of an alternative solution space, non-essentiality suggests about the robustness of the metabolic space and shows that there exists an alternative solution.

To find the allowable range of each of the reactions of the predicted chlorophyll biosynthesis pathway, we did Flux Variability analysis^67^. The Flux variability analysis (FVA) calculates the maximum and minimum allowable flux values of each of the reactions of the solution space, while satisfying the optimality of a given objective function. In brief, the method is given below:

The optimal objective value of the primary objective function (here, the sum of flux values) was computed for one flux unit of chlorophyll synthesis. Then, the primary objective function was set to this computed optimal value as an additional constraint to the linear programming problem. Then for each reaction in the solution space, the reaction flux was maximized and minimized to get the allowable range of flux values.

## Additional Information

**How to cite this article**: Chatterjee, A. and Kundu, S. Revisiting the chlorophyll biosynthesis pathway using genome scale metabolic model of *Oryza sativa japonica*. *Sci. Rep*. **5**, 14975; doi: 10.1038/srep14975 (2015).

## Supplementary Material

Supplementary Information

Supplementary Data S1

Supplementary Data S2

Supplementary Data S3

Supplementary Data S4

Supplementary Data S5

Supplementary Data S6

Supplementary Data S7

Supplementary Data S8

Supplementary Data S9

Supplementary Data S10

Supplementary Data S11

## Figures and Tables

**Table 1:** Differences between the existing model and the new model.

	Previous Model	New Model
Cytosol	Yes (1669)	Yes (1582)
Mitochondria	Yes (24)	Yes (33)
Chloroplast	Yes (42)	Yes (91)
Peroxisome	No	Yes (15)
Total No. Of Reactions	1736	1721
Total No. Of metabolites	1484	1544

The number in the bracket indicates the number of reactions in that compartment.

**Table 2:** Effect of water stress on certain chloroplastic essential enzymes of chlorophyll synthesis; essential chloroplastic, mitochondrial and cytosolic enzymes known to affect chlorophyll synthesis; the essential transporters.

Enzyme/Transporter	Compartment	EC number	Effect on enzyme activity/Pathway involved	Phenotypic effect	Reference	Simulated result to find essentiality
GDC	mito	1.4.4.2	Mutated/reduced expression	chlorosis	Engel *et al*. 2008	Yes
SHMT	mito	2.1.2.1	Mutated/reduced expression	chlorosis	Moreno *et al*. 2004	Yes
ICDH	mito	1.1.1.42	Mutated/reduced expression	chlorosis	Sienkiewicz-Porzucek, A. *et al*. 2010	Yes
AH	mito	4.2.1.3		chlorosis		Yes
ammonia tx	mito					Yes
serine tx	mito					Yes
ketoglutarate tx	mito					Yes
glycine tx	mito					Yes
CO_2_ tx	mito					Yes
citrate_tx	mito					Yes
MTHFR	cyto	1.5.1.20	In methyl cycle	Reduced chl synthesis	Wilder *et al*., 2009	Yes
MS	cyto	2.1.1.14	Decreased activity	Reduced chl synthesis	Wilder *et al*., 2009	Yes
AdoMet-Synthetase	cyto	2.5.1.6	In methyl cycle	Reduced chl synthesis	Wilder *et al*., 2009	Yes
AdoHcyase	cyto	3.3.1.1	In methyl cycle	Reduced chl synthesis	Wilder et al., 2009	Yes
CHLM	chloroplast	2.1.1.11	Decreased activity	Reduced chl synthesis	Wilder *et al*., 2009	Yes
ALAD	chloroplast	4.2.1.24	↓ By 33%	Low chl content	Dalal and Tripathy 2012	Yes
PBGD	chloroplast	2.5.1.61	↓ By 32%	Low chl content	Dalal and Tripathy 2012	Yes
CPO	chloroplast	1.3.3.3	↓ By 33%	Low chl content	Dalal and Tripathy 2012	Yes
PPO	chloroplast	1.3.3.4	↓ By 38%	Low chl content	Dalal and Tripathy 2012	Yes
Mg chelatase	chloroplast	6.6.1.1	↓ By 50%	Low chl content	Dalal and Tripathy 2012	Yes
POR	chloroplast	1.3.1.33	↓ By 38%	Low chl content	Dalal and Tripathy 2012	Yes
CHL P	chloroplast	1.3.1.83	↓ By 62-68% or if inhibited by expressing antisense RNA	Low chl content	Dalal and Tripathy 2012; Tanaka 1999	Yes
T1	chloroplast		If impaired, disrupts plastid biogenesis		Bouvier *et al*., 2006	Yes
T2	chloroplast		Required for AdoHcy transport		Hanson *et al*., 2000	Yes
Mal-Glu tx	chloroplast					Yes
Mal-2OG tx	chloroplast					Yes

ALAD, 5-aminolevulinic acid dehydratase; PBGD, Porphobilinogen deaminase; CPO, coproporphyrinogen III oxidase; PPO, protoporphyrinogen oxidase; MgCHL, Mg chelatase; POR, protochlorophyllide oxidoreductase; CHL P, geranyl geranyl reductase; T1, S-adenosylmethionine transporter; T2, Adenosyl homocysteine transporter; AH, Aconitate hydratase; GDC, glycine decarboxylase; SHMT, serine hydroxymethyltransferase; ICDH, isocitratedehydrogenase; CHLM, Mg-protoporphyrin.

IX methyltransferase; MTHFR,5,10-methylene-THF reductases; MS, methionine synthase; AdoMet-Synthetase,S-adenosylmethionine synthetase; AdoHcyase,adenosylhomocysteinase; cyto, cytosol; mito,mitochondria; chl, chlorophyll; tx, transporter.

↓ indicate decrease in the enzyme activity.
