# Supplementary material for: Revisiting the chlorophyll biosynthesis pathway using genome scale metabolic model of Oryza sativa japonica
Source: Sci Rep. 2015 Oct 7;5:14975. doi: 10.1038/srep14975 (PMC4595741; doi:10.1038/srep14975)
Supplement: Supplementary Information [file srep14975-s1.pdf]

Revisiting the chlorophyll biosynthesis pathway using genome scale metabolic model of *Oryza sativa japonica*

Authors:

First Author: Ankita Chatterjee

Academic Affiliation: Department of Biophysics, Molecular Biology and Bioinformatics; University of Calcutta

Address: 92, APC Road

City: Kolkata

Country: India

Postal Code: 700009

Email address: bioinfo.ankita21@gmail.com

Corresponding Author: Sudip Kundu[*]

Academic Affiliation: 1. Department of Biophysics, Molecular Biology and Bioinformatics; University of Calcutta

2. Center of Excellence in Systems Biology and Biomedical Engineering, TEQIP Phase-II, University of Calcutta

Address: 92, APC Road

City: Kolkata

Country: India

Postal Code: 700009

Email address: skbmbg@caluniv.ac.in

Telephone: +91-033-2350-8386

Table S1. List of reactions found to be non-plant and hence deleted from the model

Reactions deleted from the model	EC No.
RXN-7774	4.1.1.71
GLUCONATE-5-DEHYDROGENASE-RXN-(NADP)	1.1.1.69
GLUCONATE-5-DEHYDROGENASE-RXN-(NAD)	1.1.1.69
NMNAMIDOHYDRO-RXN	3.5.1.42
NAD-SYNTH-NH3-RXN	6.3.1.5
BUTYRATE-KINASE-RXN	2.7.2.7
PHOSPHATE-BUTYRYLTRANSFERASE-RXN	2.3.1.19
HYPXPRIBOSYLTRAN-RXN	#EC no. Not found #no data found for occurrence in plants
GMP-SYN-NH3-RXN	
GUANINE-DEAMINASE-RXN	3.5.4.3
DEOXYGLUCONOKIN-RXN	2.7.1.45
RXN-1981	4.2.99.21
GLUCONOLACT-RXN	3.1.1.17
KDPGALDOL-RXN	4.1.2.14
GLUCOSE-1-DEHYDROGENASE-RXN-(NADP)	1.1.1.47
PPPGPPHYDRO-RXN	3.6.1.40
GTPPYPHOSKIN-RXN	2.7.6.5
PPGPPSYN-RXN	3.1.7.2
ORNITHINE-CYCLODEAMINASE-RXN	4.3.1.12

CYSTEINE-DIOXYGENASE-RXN	1.13.11.20
RXN-974	no info
DUTP-PYROP-RXN	3.6.1.19
DEOXYINOPHOSPHOR-RXN	2.4.2.1
DEOXYRIBOSE-P-ALD-RXN	4.1.2.4
R13-RXN	3.5.4.8
D-PPENTOMUT-RXN	5.4.2.7
AICARSYN-RXN	4.3.2.2
GLYCINE-FORMIMINOTRANSFERASE-RXN	2.1.2.4
R63-RXN	3.5.2.-
AIRCARBOXY-RXN	4.1.1.21
R128-RXN	3.5.1.-
R127-RXN	no info
R62-RXN	no info
IMPCYCLOHYDROLASE-RXN	3.5.4.10
4-DIAMINOPENTANOATE-DEHYDROGENASE-RXN-(NAD)	1.4.1.12
ARGININE-2-MONOOXYGENASE-RXN	1.13.12.1
4-DIAMINOPENTANOATE-DEHYDROGENASE-RXN-(NADP)	1.4.1.12
ACETYLORNDEACET-RXN	3.5.1.16
GUANIDINOBUTYRASE-RXN	3.5.3.7
RXN-22	4.3.2.1
PYRUVOXID-RXN	1.2.5.1

RXN-8089	1.2.3.9
CYSTATHIONINE-BETA-SYNTHASE-RXN	4.2.1.22
TREHALA-RXN	3.2.1.28
PYRUFLAVREDUCT-RXN	1.2.7.1
R11-RXN	2.8.3.8
ACETALD-DEHYDROG-RXN	1.2.1.10
HOMOSERINE-O-ACETYLTRANSFERASE-RXN	2.3.1.31
THREONINE-ALDOLASE-RXN	4.1.2.5
AICARTRANSFORM-RXN	2.1.2.3
RXN-9	2.1.3.3
RXN-10	6.3.4.5
RXN-34	3.5.3.1
CITRULLINASE-RXN	3.5.1.20
MYO-INOSITOL-1(OR-4)-MONOPHOSPHATASE-RXN	3.1.3.25

Table S2. (a) Detail of all the reactions and transporters involved in one of the possible chlorophyll synthesis pathways.

Reaction Name	Reaction
mit_AconDHatase	1.0 Citrate <> 1.0 CisAconitate
chl_FPPSYNRXN	1.0 GERANYL-PP_str + 1.0 delta(3)-isopentenyl-pp_str -> 1.0 FARNESYL-PP_str +
chl_PROTOPORGENOXIRXN	2.0 PROTOPORPHYRINOGEN_str + 3.0 O2_str -> 2.0 PROTOPORPHYRIN_IX_str
chl_UROGENIIISYNRXN	1.0 HYDROXYMETHYLBILANE_str -> 1.0 UROPORPHYRINOGEN-III_str
1.5.1.20-RXN-(NADP)	1.0 NADP + 1.0 5-METHYL-THF <> 1.0 NADPH + 1.0 METHYLENE-THF
chl_PYR_tx	1.0 PYRUVATE <> 1.0 PYR_str
ACETATE--COA-LIGASE-RXN	1.0 CO-A + 1.0 ATP + 1.0 ACET -> 1.0 AMP + 1.0 PPI + 1.0 ACETYL-COA
chl_CDPKINRXN	1.0 ATP_str + 1.0 CDP_str -> 1.0 ADP_str + 1.0 CTP_str
chl_GAP_tx	1.0 Pi_str + 1.0 GAP <> 1.0 Pi + 1.0 GAP_str
chl_OHMETHYLBILANESYNRXN	4.0 PORPHOBILINOGEN_str -> 1.0 HYDROXYMETHYLBILANE_str + 4.0 AMM
PEPDEPHOS-RXN	1.0 PHOSPHO-ENOL-PYRUVATE + 1.0 ADP -> 1.0 PYRUVATE + 1.0 ATP
chl_LightNonCyc	4.5 ADP_str + 3.5 NADP_str + 14.0 Photon + 4.5 Pi_str -> 4.5 ATP_str + 3.5 NADPH
chl_CO2_tx	1.0 CARBON-DIOXIDE -> 1.0 CO2_str
chl_RXN1F72	1.0 DIVINYL-PROTOCHLOROPHYLLIDE-A_str + 1.0 NADPH_str -> 1.0 MONO-
ADENYL-KIN-RXN	1.0 AMP + 1.0 ATP <> 2.0 ADP
CO2_tx	1 x_CO2 <> 1 CARBON-DIOXIDE
chl_LightCyc	6.0 ADP_str + 14.0 Photon + 6.0 Pi_str -> 6.0 ATP_str
O2_tx	1 x_O2 <> 1 OXYGEN-MOLECULE
chl_G3Pdh	1.0 BPGA_str + 1.0 NADPH_str <> 1.0 NADP_str + 1.0 GAP_str + 1.0 Pi_str
chl_PyrNADPH	1.0 NADPH_str + 1.0 PYR_str + 1.0 CO2_str -> 1.0 NADP_str + 1.0 MAL_str
INORGPYROPHOSPHAT-RXN	1.0 PPI -> 2.0 Pi
mit_Complex_III	1.0 QH2 + 2.0 Cyt_ox + 2.0 Proton -> 1.0 Q + 2.0 Cyt_red
chl_RXN0882	1.0 NADP_str + 1.0 HYDROXY-METHYL-BUTENYL-DIP_str <> 1.0 NADPH_str
mit_IsoCitDH	1.0 NAD_mit + 1.0 IsoCitrate -> 1.0 NAD_mitH + 1.0 mit_CO2 + 1.0 AlphaKG + 2.0
RXN0-5114	1.0 3-P-SERINE <> 1.0 Pi + 1.0 SER
chl_X5Piso	1.0 X5P_str <> 1.0 Ru5P_str
chl_RXN1F66	1.0 PHYTYL-PYROPHOSPHATE_str + 1.0 CHLOROPHYLLIDE-A_str -> 1.0 CHL
RXN66-3	1.0 NAD + 1.0 ACETALD -> 1.0 NADH + 1.0 ACET

chl_Ru5Pk	1.0 ATP_str + 1.0 Ru5P_str -> 1.0 RuBP_str + 1.0 ADP_str
chl_MalGLT_tx	1.0 GLT + 1.0 MAL_str <> 1.0 GLT_str + 1.0 MAL
chl_TPI	1.0 GAP_str <> 1.0 DHAP_str
mit_Cit_tx	1.0 CIT <> 1.0 Citrate
chl_R5Piso	1.0 R5P_str <> 1.0 Ru5P_str
CHL_A_tx	x_CHLOROPHYLLA <> 1 CHLOROPHYLL-A_str
MALATE-DEH-RXN	1.0 MAL + 1.0 NAD <> 1.0 NADH + 1.0 OXALACETIC_ACID
chl_RXN0302	1.0 2-PHOSPHO-4-CYTIDINE-5-DIPHOSPHO-2-C-MET_str <> 1.0 CMP_str + 1.0 2
PSERTRANSAM-RXN	1.0 3-P-SERINE + 1.0 2-KETOGLUTARATE <> 1.0 3-P-HYDROXYPYRUVATE +
chl_Eno	1.0 PGA2_str <> 1.0 PEP_str
mit_SER_tx	1.0 SER <> 1.0 mit_SER
chl_UROGENDECARBOXRXN	1.0 UROPORPHYRINOGEN-III_str -> 1.0 COPROPORPHYRINOGEN_III_str + 4.0
mit_O2_tx	1.0 OXYGEN-MOLECULE <> 1.0 mit_O2
chl_DXSRXN	1.0 GAP_str + 1.0 PYR_str -> 1.0 DEOXYXYLULOSE-5P_str + 1.0 CO2_str
chl_PPase	1.0 PPI_str -> 2.0 Pi_str
chl_RXN1F10	1.0 MONO-VINYL-PROTOCHLOROPHYLLIDE-A_str + 1.0 NADPH_str -> 1.0 NA
chl_PGlyM	1.0 PGA_str <> 1.0 PGA2_str
ATP-CITRATE-(PRO-S-)-LYASE-RXN	1.0 Pi + 1.0 OXALACETIC_ACID + 1.0 ACETYL-COA + 1.0 ADP -> 1.0 CO-A + 1.
chl_61118RXN	1.0 GLT-tRNAs_str + 1.0 ATP_str + 1.0 GLT_str -> 1.0 AMP_str + 1.0 Charged-GLT
mit_GCVMULTI	1.0 NAD_mit + 1.0 mit_GLY + 1.0 mit_THF -> 1.0 NAD_mitH + 1.0 mit_CO2 + 1.0
chl_GLUTRNAREDUCTRXN	1.0 GLT-tRNAs_str + 1.0 NADP_str + 1.0 GLUTAMATE-1-SEMIALDEHYDE_str <
chl_GLUTAMATESYN	1.0 2-KETOGLUTARATE_str + 1.0 NADPH_str + 1.0 GLN_str -> 1.0 NADP_str + 2
mit_AconHydr	1.0 CisAconitate <> 1.0 IsoCitrate
chl_RXN7660	1.0 NADPH_str + 1.0 CPD-7003_str <> 1.0 PHYTYL-PYROPHOSPHATE_str + 1.0
chl_PORPHOBILSYNTHRXN	2.0 5-AMINO-LEVULINATE_str -> 1.0 PORPHOBILINOGEN_str
chl_GSAAMINOTRANSRXN	1.0 GLUTAMATE-1-SEMIALDEHYDE_str <> 1.0 5-AMINO-LEVULINATE_str
T2	1.0 ADENOSYL-HOMO-CYS_str <> 1.0 ADENOSYL-HOMO-CYS
T1	1.0 S-ADENOSYLMETHIONINE <> 1.0 S-ADENOSYLMETHIONINE_str
chl_TKL1	1.0 GAP_str + 1.0 F6P_str <> 1.0 X5P_str + 1.0 E4P_str
chl_TKL2	1.0 S7P_str + 1.0 GAP_str <> 1.0 X5P_str + 1.0 R5P_str
chl_27760RXN	1.0 CTP_str + 1.0 2-C-METHYL-D-ERYTHRITOL-4-PHOSPHATE_str <> 1.0 4-CY
chl_Photon_tx	x_Photon -> Photon

chl_MalOxAc_tx	1.0 OXALACETIC_ACID + 1.0 MAL_str <> 1.0 OAA_str + 1.0 MAL
PYRUVATE-DECARBOXYLASE-RXN	1.0 PYRUVATE -> 1.0 CARBON-DIOXIDE + 1.0 ACETALD
chl_FBPase	1.0 FBP_str -> 1.0 F6P_str + 1.0 Pi_str
HOMOCYSMETB12-RXN	1.0 HOMO-CYS + 1.0 5-METHYL-THF -> 1.0 THF + 1.0 MET
chl_RXNMGPROTOPORPHYRINMETHYLESTERSYN	1.0 S-ADENOSYLMETHIONINE_str + 1.0 MG-PROTOPORPHYRIN_str -> 1.0 MG
2PGADEHYDRAT-RXN	1.0 2-PG <> 1.0 PHOSPHO-ENOL-PYRUVATE
chl_RXN7659	1.0 NADPH_str + 1.0 CPD-7002_str <> 1.0 NADP_str + 1.0 CPD-7003_str
chl_RXN7658	1.0 GERANYLGERANYL-PP_str + 1.0 NADPH_str <> 1.0 NADP_str + 1.0 CPD-70
chl_RXN0884NADP	1.0 NADPH_str + 1.0 HYDROXY-METHYL-BUTENYL-DIP_str <> 1.0 NADP_str +
PGLYCDEHYDROG-RXN	1.0 G3P + 1.0 NAD <> 1.0 3-P-HYDROXYPYRUVATE + 1.0 NADH
chl_CMPKIRXN	1.0 ATP_str + 1.0 CMP_str <> 1.0 ADP_str + 1.0 CDP_str
GLYOHMETRANS-RXN	1.0 METHYLENE-THF + 1.0 GLY <> 1.0 THF + 1.0 SER
mit_CO2_tx	1.0 CARBON-DIOXIDE <> 1.0 mit_CO2
3PGAREARR-RXN	1.0 G3P <> 1.0 2-PG
ADENOSINE-KINASE-RXN	1.0 ATP + 1.0 ADENOSINE -> 1.0 AMP + 1.0 ADP
chl_O2_tx	1.0 OXYGEN-MOLECULE <> 1.0 O2_str
chl_MalDH	1.0 NADP_str + 1.0 MAL_str <> 1.0 OAA_str + 1.0 NADPH_str
chl_DXPREDISOMRXN	1.0 NADP_str + 1.0 2-C-METHYL-D-ERYTHRITOL-4-PHOSPHATE_str <> 1.0 DE
chl_PyrK	1.0 ADP_str + 1.0 PEP_str -> 1.0 ATP_str + 1.0 PYR_str
chl_GLUTAMINESYN	1.0 ATP_str + 1.0 GLT_str + 1.0 AMMONIA_str -> 1.0 ADP_str + 1.0 GLN_str + 1.0
chl_Mal2OG_tx	1.0 MAL_str + 1.0 2-KETOGLUTARATE <> 1.0 2-KETOGLUTARATE_str + 1.0 M
chl_Ald1	1.0 DHAP_str + 1.0 GAP_str <> 1.0 FBP_str
chl_Ald2	1.0 DHAP_str + 1.0 E4P_str <> 1.0 SBP_str
ex_ammonia_tx	x_NH3 -> AMMONIA_str
mit_AKG_tx	1.0 2-KETOGLUTARATE <> 1.0 AlphaKG
chl_271148RXN	1.0 ATP_str + 1.0 4-CYTIDINE-5-DIPHOSPHO-2-C_str <> 1.0 ADP_str + 1.0 2-PHO
chl_RXN1F20	1.0 ATP_str + 1.0 PROTOPORPHYRIN_IX_str -> 1.0 ADP_str + 1.0 MG-PROTOPO
chl_RXN01461	1.0 O2_str + 1.0 COPROPORPHYRINOGEN_III_str <> 1.0 PROTOPORPHYRINOG
1.2.1.9-RXN	1.0 NADP + 1.0 GAP -> 1.0 NADPH + 1.0 G3P
chl_FARNESYLTRANSTRANSFERASERXN	1.0 FARNESYL-PP_str + 1.0 delta(3)-isopentenyl-pp_str <> 1.0 GERANYLGERANY
mit_GLYOHMETRANS	1.0 mit_GLY + 1.0 mit_METHYLENE-THF <> 1.0 mit_THF + 1.0 mit_SER
chl_SBPase	1.0 SBP_str -> 1.0 S7P_str + 1.0 Pi_str

chl_ADENYLKINRXN	1.0 AMP_str + 1.0 ATP_str <> 2.0 ADP_str
ADENOSYLHOMOCYSTEINASE-RXN	1.0 ADENOSYL-HOMO-CYS <> 1.0 HOMO-CYS + 1.0 ADENOSINE
S-ADENMETSYN-RXN	1.0 MET + 1.0 ATP -> 1.0 PPI + 1.0 Pi + 1.0 S-ADENOSYLMETHIONINE
mit_AOX	2.0 QH2 + 1.0 mit_O2 -> 2.0 Q
chl_PGK	1.0 ATP_str + 1.0 PGA_str <> 1.0 ADP_str + 1.0 BPGA_str
chl_Rubisco	1.0 RuBP_str + 1.0 CO2_str -> 2.0 PGA_str
chl_RXN5282	1.0 MG-PROTOPORPHYRIN-MONOMETHYL-ESTER_str + 1.0 NADPH_str + 1.0
chl_RXN5283	1.0 NADPH_str + 1.0 O2_str + 1.0 13-HYDROXY-MAGNESIUM-PROTOPORP_str
chl_RXN5284	1.0 NADPH_str + 1.0 O2_str + 1.0 131-OXO-MAGNESIUM-PROTOPORPHYRIN-I
mit_Complex_V	1.0 Pi + 1.0 ADP -> 4.0 Proton + 1.0 ATP
chl_GPPSYNRXN	1.0 CPD-4211_str + 1.0 delta(3)-isopentenyl-pp_str -> 1.0 PPI_str + 1.0 GERANYL-P
chl_ISPH2RXNNADP	1.0 NADPH_str + 1.0 HYDROXY-METHYL-BUTENYL-DIP_str <> 1.0 NADP_str +
mit_ammonia_tx	1.0 mit_AMMONIA -> 1.0 AMMONIA_str
mit_Complex_I	1.0 Q + 1.0 NAD_mitH + 5.0 Proton -> 1.0 QH2 + 1.0 NAD_mit
mit_Complex_IV	1.0 mit_O2 + 8.0 Proton + 4.0 Cyt_red -> 4.0 Cyt_ox
mit_GLY_tx	1.0 GLY <> 1.0 mit_GLY

Chl, chloroplast; mit, mitochondria; str, stroma; tx, transporter; x_, external

(b) The essential reactions, enzymes and genes.

**Reaction Name**	**Reaction**	**Enzyme**	**EC No.**	**Locus**
chl_FPPSYNRXN	1.0 GERANYL-PP_str + 1.0 delta(3)-isopentenyl-pp_str -> 1.0 FARNESYL-PP_str + 1.0 PPI_str	farnesyl diphosphate synthase	2.5.1.10	LOC_Os01g58790
chl_PROTOPORGENOXIRXN	2.0 PROTOPORPHYRINOGEN_str + 3.0 O2_str -> 2.0 PROTOPORPHYRIN_IX_str	protoporphyrinogen oxidase	1.3.3.4	LOC_OS10G38850.1, LOC_OS04G57560,LOC_Os01g18320.1
chl_CDPKINRXN	1.0 ATP_str + 1.0 CDP_str ->	nucleoside-	2.7.4.6	LOC_Os12g36194,LOC_Os07g30970,LOC_Os02g35700,LOC_Os10g

	1.0 ADP_str + 1.0 CTP_str	diphosphate kinase		41410,LOC_Os05g51700
chl_OHMETHYLBILANESYNRXN	4.0 PORPHOBILINOGEN_str -> 1.0 HYDROXYMETHYLBILANE_str + 4.0 AMMONIA_str	hydroxymethylbilane synthase	2.5.1.61	LOC_Os02g07230.1
chl_LightNonCyc	4.5 ADP_str + 3.5 NADP_str + 14.0 Photon + 4.5 Pi_str -> 4.5 ATP_str + 3.5 NADPH_str + 1.75 O2_str			
chl_RXN1F72	1.0 DIVINYL-PROTOCHLOROPHYLLIDE-A_str + 1.0 NADPH_str -> 1.0 MONO-VINYL-PROTOCHLOROPHYLLIDE-A_str + 1.0 NADP_str	divinyl chlorophyllide a 8-vinyl-reductase	1.3.1.75	LOC_Os03g22780.1
chl_G3Pdh	1.0 BPGA_str + 1.0 NADPH_str <> 1.0 NADP_str + 1.0 GAP_str + 1.0 Pi_str	glyceraldehyde-3-phosphate dehydrogenase	1.2.1.59	LOC_Os06g45590,LOC_Os04g38600,LOC_Os04g40950,LOC_Os08g03290,LOC_Os02g07490,LOC_Os02g38920,LOC_Os03g03720,
chl_PyrNADPH	1.0 NADPH_str + 1.0 PYR_str + 1.0 CO2_str -> 1.0 NADP_str + 1.0 MAL_str			
chl_X5Piso	1.0 X5P_str <> 1.0 Ru5P_str			
chl_RXN1F66	1.0 PHYTYL-PYROPHOSPHATE_str + 1.0 CHLOROPHYLLIDE-A_str -> 1.0 CHLOROPHYLL-A_str + 1.0 PPI_str	chlorophyll synthetase	2.5.1.62	LOC_OS05G28200.1

chl_RXN0882	1.0 NADP_str + 1.0 HYDROXY-METHYL-BUTENYL-DIP_str <> 1.0 NADPH_str + 1.0 2C-METH-D-ERYTHRITOL-CYCLODIPHOSPHATE_str	4-hydroxy-3-methylbut-2-en-1-yl diphosphate synthase	1.17.7.1	LOC_Os02g39160.1
chl_Ru5Pk	1.0 ATP_str + 1.0 Ru5P_str -> 1.0 RuBP_str + 1.0 ADP_str	phosphoribulokinase	2.7.1.19	LOC_Os04g21110,LOC_Os04g50880,LOC_Os02g47020,LOC_Os12g40880,LOC_Os08g41790
chl_R5Piso				
chl_RXN0302	1.0 2-PHOSPHO-4-CYTIDINE-5-DIPHOSPHO-2-C-MET_str <> 1.0 CMP_str + 1.0 2C-METH-D-ERYTHRITOL-CYCLODIPHOSPHATE_str	2-C-methyl-D-erythritol 2,4-cyclodiphosphate synthase	4.6.1.12	LOC_Os02g45660
chl_UROGENDECARBOXRXN	1.0 UROPORPHYRINOGEN-III_str -> 1.0 COPROPORPHYRINOGEN_III_str + 4.0 CO2_str	uroporphyrinogen decarboxylase	4.1.1.37	LOC_OS03G22060.1,LOC_OS03G21900.1 ,LOC_OS01G43390.1
chl_DXSRXN	1.0 GAP_str + 1.0 PYR_str -> 1.0 DEOXYXYLULOSE-5P_str + 1.0 CO2_str	1-deoxy-D-xylulose-5-phosphate synthase	2.2.1.7	LOC_Os05g33840.1,LOC_Os07g09190.1
chl_PPase	1.0 PPI_str -> 2.0 Pi_str			
chl_RXN1F10	1.0 MONO-VINYL-PROTOCHLOROPHYLLIDE-A_str + 1.0 NADPH_str -> 1.0 NADP_str + 1.0 CHLOROPHYLLIDE-A_str	protochlorophyllide oxidoreductase (POR)	1.3.1.33	LOC_Os10g35370.1,LOC_Os04g58200.1

chl_61117RXN	1.0 tRNAGlu_str + 1.0 ATP_str + 1.0 GLT_str -> 1.0 AMP_str + 1.0 L-glutamyl-tRNAGlu_str + 1.0 PPI_str	Glutamate-tRNA ligase	6.1.1.17	LOC_Os01g09000.1, LOC_Os05g08990.1
chl_GLUTRNAREDUCTRXN	1.0 L-glutamyl-tRNAGlu_str + 1.0 NADP_str + 1.0 glutamate-1-semialdehyde_str <> 1.0 NADPH_str + 1.0 tRNA-Glu_str	glutamyl-tRNA reductase (GluTR)	1.2.1.70	LOC_Os10g35840.1
chl_RXN7660	1.0 NADPH_str + 1.0 geranylgeranyl-diphosphate_str <> 1.0 phytyl-diphosphate_str + 1.0 NADP_str	geranyl geranyl reductase	1.3.1.83	LOC_Os02g51080.1
chl_PORPHOBILSYNTHRXN	2.0 5-AMINO-LEVULINATE_str -> 1.0 PORPHOBILINOGEN_str	delta-aminolevulinic acid dehydratase	4.2.1.24	LOC_Os06g49110.1
chl_GSAAMINOTRANSRXN	1.0 GLUTAMATE-1-SEMIALDEHYDE_str <> 1.0 5-AMINO-LEVULINATE_str	glutamate-1-semialdehyde 2,1-aminomutase	5.4.3.8	LOC_Os03g44150.1 ,LOC_Os08g41990.1
chl_TKL1	1.0 GAP_str + 1.0 F6P_str <> 1.0 X5P_str + 1.0 E4P_str	transketolase	2.2.1.1	LOC_Os06g04270.1
chl_TKL2	1.0 GAP_str + 1.0 F6P_str <> 1.0 X5P_str + 1.0 E4P_str	transketolase	2.2.1.1	LOC_Os06g04270.1
chl_27760RXN	1.0 CTP_str + 1.0 2-C-METHYL-D-ERYTHRITOL-4-PHOSPHATE_str <> 1.0 4-CYTIDINE-5-DIPHOSPHO-2-C_str + 1.0 PPI_str	2-C-methyl-D-erythritol 4-phosphate cytidylyltransferase	2.7.7.60	LOC_Os01g66360

chl_RXNMGPROTOPORPHYRINMETHYLESTERSYN	1.0 S-ADENOSYLMETHIONINE_str + 1.0 MG-PROTOPORPHYRIN_str -> 1.0 MG-PROTOPORPHYRIN-MONOMETHYL-ESTER_str + 1.0 ADENOSYL-HOMO-CYS_str	magnesium protoporphyrin IX methyltransferase (ChlM)	2.1.1.11	LOC_OS06G04150.1
chl_RXN7659	1.0 NADPH_str + 1.0 geranylgeranyl-diphosphate_str <> 1.0 NADP_str + 1.0 phytyl-diphosphate _str	geranyl geranyl reductase (chlP)	1.3.1.83	LOC_Os02g51080
chl_RXN7658	1.0 NADPH_str + 1.0 geranylgeranyl-diphosphate_str <> 1.0 NADP_str + 1.0 phytyl-diphosphate _str	geranyl geranyl reductase (chlP)	1.3.1.83	LOC_Os02g51080
chl_CMPKIRXN	1.0 ATP_str + 1.0 CMP_str <> 1.0 ADP_str + 1.0 CDP_str	cytidine monophosphate kinase	2.7.4.14	
chl_DXPREDISOMRXN	1.0 NADP_str + 1.0 2-C-METHYL-D-ERYTHRITOL-4-PHOSPHATE_str <> 1.0 DEOXYXYLULOSE-5P_str + 1.0 NADPH_str	1-deoxy-D-xylulose-5-phosphate reductoisomerase (DXR)	1.1.1.267	LOC_Os01g01710.1
chl_GLUTAMINESYN	1.0 ATP_str + 1.0 GLT_str + 1.0 AMMONIA_str -> 1.0 ADP_str + 1.0 GLN_str + 1.0 Pi_str	glutamine synthetase	6.3.1.2	LOC_Os03g12290.1 ; LOC_Os03g50490.1

chl_Ald2	1.0 DHAP_str + 1.0 E4P_str <> 1.0 SBP_str			
chl_271148RXN	1.0 ATP_str + 1.0 4-CYTIDINE-5-DIPHOSPHO-2-C_str <> 1.0 ADP_str + 1.0 2-PHOSPHO-4-CYTIDINE-5-DIPHOSPHO-2-C-MET_str	4-(cytidine 5'-diphospho)-2-C-methyl-D-erythritol kinase (CDPMEK)	2.7.1.148	LOC_Os01g58790
chl_RXN1F20	1.0 ATP_str + 1.0 PROTOPORPHYRIN_IX_str -> 1.0 ADP_str + 1.0 MG-PROTOPORPHYRIN_str + 1.0 Pi_str	magnesium chelatase (ChlH)	6.6.1.1	LOC_OS07G46310.1,LOC_OS08G31870.1, LOC_OS10G30580.1
chl_RXN01461	1.0 O2_str + 1.0 COPROPORPHYRINOGEN_III_str <> 1.0 PROTOPORPHYRINOGEN_str + 2.0 CO2_str	coproporphyrinogen III oxidase (CPO)	1.3.3.3	LOC_Os04g52130
chl_FARNESYLTRANSTRANSFERASERXN	1.0 farnesyl-diphosphate_str + 1.0 isopentenyl-diphosphate_str <> 1.0 geranylgeranyl-diphosphate_str + 1.0 diphosphate_str	geranylgeranyl diphosphate synthase	2.5.1.29	LOC_Os09g33930.1 ,LOC_Os01g53600.1 ,LOC_Os07g39270.1 ,LOC_Os02g44780.1 , LOC_Os01g14630.1
chl_SBPase	1.0 SBP_str -> 1.0 S7P_str + 1.0 Pi_str	sedoheptulose-bisphosphatase	3.1.3.37	
chl_ADENYLKINRXN	1.0 AMP_str + 1.0 ATP_str <> 2.0 ADP_str	adenylate kinase	2.7.4.3	LOC_Os08g19140.1 ,LOC_Os08g02540.1 ,LOC_Os08g01770.1,LOC_Os07g43170.1 , LOC_Os06g10200.1 ,LOC_Os06g02000.1,LOC_Os04g57540.1

				,LOC_Os04g01530.1,LOC_Os03g03820.1 , LOC_Os02g53790.1,LOC_Os12g13380.1
chl_UROGENIIISYNRXN	1.0 HYDROXYMETHYLBILANE_str -> 1.0 UROPORPHYRINOGEN-III_str	uroporphyrinogen III synthase	4.2.1.75	LOC_Os03g08730.1
chl_PGK	1.0 ATP_str + 1.0 PGA_str <> 1.0 ADP_str + 1.0 BPGA_str	phosphoglycerate kinase	2.7.2.3	LOC_Os06g45710.1 , LOC_Os05g41640.1 ,LOC_Os02g07260.1,LOC_Os06g45710.1
chl_Rubisco	1.0 RuBP_str + 1.0 CO2_str -> 2.0 PGA_str	Ribulose-bisphosphate carboxylase	4.1.1.39	LOC_Os01g58020,LOC_Os10g21268,LOC_Os12g10580,LOC_Os11g32770,LOC_Os05g35330,LOC_Os02g05830,LOC_Os12g17600,LOC_Os12g19381,LOC_Os12g19470,LOC_Os09g24530
chl_RXN5282	1.0 MG-PROTOPORPHYRIN-MONOMETHYL-ESTER_str + 1.0 NADPH_str + 1.0 O2_str -> 1.0 NADP_str + 1.0 13-HYDROXY-MAGNESIUM-PROTOPORP_str	MgPME cyclase	1.14.13.81	LOC_Os01g17170
chl_RXN5283	1.0 MG-PROTOPORPHYRIN-MONOMETHYL-ESTER_str + 1.0 NADPH_str + 1.0	MgPME cyclase	1.14.13.81	LOC_Os01g17170

	O2_str -> 1.0 NADP_str + 1.0 13-HYDROXY-MAGNESIUM-PROTOPORP_str			
chl_RXN5284	1.0 MG-PROTOPORPHYRIN-MONOMETHYL-ESTER_str + 1.0 NADPH_str + 1.0 O2_str -> 1.0 NADP_str + 1.0 13-HYDROXY-MAGNESIUM-PROTOPORP_str	MgPME cyclase	1.14.13.81	LOC_Os01g17170
chl_GPPSYNRXN	1.0 dimethylallyl diphosphate_str + 1.0 delta(3)-isopentenyl-pp_str -> 1.0 PPI_str + 1.0 geranyl-diphosphate_str	Dimethylallyltransferase/GPP synthase	2.5.1.1	LOC_Os12g17320.1,LOC_Os12g08070.1,LOC_Os08g29970.1,LOC_Os08g29910.1,LOC_Os08g23320.1,LOC_Os08g09370.1,LOC_Os06g46450.1,LOC_Os05g50550.1,LOC_Os05g23950.1, LOC_Os04g14190.1, LOC_Os04g14110.1,LOC_Os03g09060.1,LOC_Os02g39290.1, LOC_Os01g50050.1, LOC_Os01g50760.1
mit_AconDHatase	1.0 Citrate <> 1.0 CisAconitate	aconitate hydratase	4.2.1.3	LOC_Os06g19960,LOC_Os08g09200,LOC_Os03g04410
mit_IsoCitDH	1.0 NAD_mit + 1.0 IsoCitrate -> 1.0 NAD_mitH + 1.0 mit_CO2 + 1.0 AlphaKG	Isocitrate dehydrogenase	1.1.1.42	LOC_Os01g46610.1, LOC_Os01g14580.1,LOC_Os05g49760.1, LOC_Os04g42920.1
mit_GCVMULTI	1.0 NAD_mit + 1.0 mit_GLY + 1.0 mit_THF <> 1.0 NAD_mitH + 1.0 mit_CO2 +	glycine decarboxylase (GDC)	1.4.4.2	LOC_Os06g45670.1; LOC_Os06g45670.1; LOC_Os06g45670.

	1.0 mit_AMMONIA + 1.0 mit_METHYLENE-THF			
mit_AconHydr	1.0 CisAconitate <> 1.0 IsoCitrate	aconitate hydratase	4.2.1.3	LOC_Os06g19960,LOC_Os08g09200,LOC_Os03g04410
mit_GLYOHMETRANS	1.0 mit_GLY + 1.0 mit_METHYLENE-THF <> 1.0 mit_THF + 1.0 mit_SER	5,10-methylenetetrahydrofolate:glycine hydroxymethyltransferase (SHMT)	2.1.2.1	LOC_Os03g52840.1,LOC_Os05g35440.1,LOC_Os11g26860
RXN0-5114	1.0 3-P-SERINE <> 1.0 Pi + 1.0 SER	3-phosphoserine phosphatase	3.1.3.3	LOC_Os11g41160.1 ,LOC_Os12g31820.1
PSERTRANSAM-RXN	1.0 3-P-SERINE + 1.0 2-KETOGLUTARATE <> 1.0 3-P-HYDROXYPYRUVATE + 1.0 GLT	phosphoserine transaminase	2.6.1.52	LOC_Os03g06200.1
HOMOCYSMETB12-RXN	1.0 HOMO-CYS + 1.0 5-METHYL-THF -> 1.0 THF + 1.0 MET	5-methyltetrahydropteroyltriglutamate-homocysteine S-methyltransferase	2.1.1.14	LOC_Os03g63330.1
PGLYCDEHYDROG-RXN	1.0 G3P + 1.0 NAD <> 1.0 3-P-HYDROXYPYRUVATE + 1.0 NADH	D-3-phosphoglycerate dehydrogenase	1.1.1.95	LOC_Os07g15970.1 ,LOC_Os08g34720.1 ,LOC_Os06g44460.1 ,LOC_Os04g55720.1
GLYOHMETRANS-RXN	Gly + Methylene-THF <> THF + Ser	5,10-methylenetetrahydrofolate:glycine hydroxymethyltransferase	2.1.2.1	LOC_Os03g52840.1,LOC_Os05g35440.1,LOC_Os11g26860
ATP-CITRATE-(PRO-S-)-LYASE-	1.0 Pi + 1.0 OXALACETIC_ACID + 1.0	ATP-citrate lyase	2.3.3.8	LOC_Os01g19450

RXN	ACETYL-COA + 1.0 ADP -> 1.0 CO-A + 1.0 ATP + 1.0 CIT			
ADENOSYLHOMOCYSTEINASE-RXN	1.0 ADENOSYL-HOMO-CYS <> 1.0 HOMO-CYS + 1.0 ADENOSINE	adenosylhomocysteinase	3.3.1.1	LOC_Os11g26850.1 ,, LOC_Os04g46450.1
S-ADENMETSYN-RXN	1.0 MET + 1.0 ATP -> 1.0 PPI + 1.0 Pi + 1.0 S-ADENOSYLMETHIONINE	S-adenosylmethionine synthetase	2.5.1.6	LOC_Os07g29440.1 ,LOC_Os05g04510.1 ,LOC_Os01g10940.1
ACETATE--COA-LIGASE-RXN	1.0 CO-A + 1.0 ATP + 1.0 ACET -> 1.0 AMP + 1.0 PPI + 1.0 ACETYL-COA	acetate-CoA ligase	6.2.1.1	LOC_Os02g32490.1, LOC_Os04g33190.1
PYRUVATE-DECARBOXYLASE-RXN	1.0 PYRUVATE -> 1.0 CARBON-DIOXIDE + 1.0 ACETALD	Indole-3-pyruvic acid decarboxylase	4.1.1.1	LOC_Os03g18220.1 , LOC_Os05g39320.1 , LOC_Os05g39310.1 , LOC_Os07g49250.1
2PGADEHYDRAT-RXN	1.0 2-PG <> 1.0 PHOSPHO-ENOL-PYRUVATE	phosphopyruvate hydratase	4.2.1.11	LOC_Os10g08550.1 , LOC_Os06g04510.1 , LOC_Os03g14450.1
MALATE-DEH-RXN	1.0 MAL + 1.0 NAD <> 1.0 NADH + 1.0 OXALACETIC_ACID	Malate dehydrogenase	1.1.1.37	LOC_Os12g43630.1, LOC_Os10g33800.1, LOC_Os08g33720.1, LOC_Os01g61380.1, LOC_Os01g46070.1, LOC_Os04g46560.1
3PGAREARR-RXN	1.0 G3P <> 1.0 2-PG	2,3bisphosphoglycer-ate-independent phosphoglycerate mutase	5.4.2.1	LOC_Os05g40420.1, LOC_Os03g21260.1, LOC_Os01g60190.1,
ADENOSINE-KINASE-RXN	1.0 ATP + 1.0 ADENOSINE -> 1.0 AMP + 1.0 ADP	Adenosine kinase	2.7.1.20	LOC_Os06g12600.1, LOC_Os04g43750.1, LOC_Os02g41590.1
RXN66-3	1.0 NAD + 1.0 ACETALD -> 1.0 NADH + 1.0 ACET	Aldehyde dehydrogenase	1.2.1.3	LOC_Os02g43280.1 LOC_Os02g43194.1 LOC_Os04g45720.1 LOC_Os11g08300.1

1.2.1.9-RXN	1.0 NADP + 1.0 GAP -> 1.0 NADPH + 1.0 G3P	glyceraldehyde-3-phosphate dehydrogenase	1.2.1.9	LOC_Os07g48920.1<br>LOC_Os02g07760.1<br>LOC_Os01g40860.1<br>LOC_Os03g03720.1
## transporters				
mit_AKG_tx	1.0 2-KETOGLUTARATE <> 1.0 AlphaKG			
mit_GLY_tx	1.0 GLY <> 1.0 mit_GLY			
mit_SER_tx	1.0 SER <> 1.0 mit_SER			
mit_Cit_tx	1.0 CIT <> 1.0 Citrate			
mit_CO2_tx	1.0 CARBON-DIOXIDE <> 1.0 mit_CO2			
mit_ammonia_tx	1.0 mit_AMMONIA -> 1.0 AMMONIA_str			
mit_O2_tx	1.0 OXYGEN-MOLECULE <> 1.0 mit_O2			
chl_CO2_tx	1.0 CARBON-DIOXIDE -> 1.0 CO2_str			
chl_MalGLT_tx	1.0 GLT + 1.0 MAL_str <> 1.0 GLT_str + 1.0 MAL			
T2	1.0 ADENOSYL-HOMO-CYS_str <> 1.0 ADENOSYL-HOMO-CYS			
T1	1.0 S-ADENOSYLMETHIONINE			

	<> 1.0 S-ADENOSYLMETHIONINE_str
chl_Photon_tx	x_Photon -> 1.0 Photon
chl_O2_tx	1.0 OXYGEN-MOLECULE <> 1.0 O2_str
chl_Mal2OG_tx	1.0 MAL_str + 1.0 2-KETOGLUTARATE <> 1.0 2-KETOGLUTARATE_str + 1.0 MAL
CO2_tx	x_CO2 <> 1.0 CARBON-DIOXIDE
O2_tx	x_O2 <> 1.0 OXYGEN-MOLECULE
CHL_A_tx	x_CHLOROPHYLL-A <> 1.0 CHLOROPHYLL-A_str

Chl, chloroplast; mit, mitochondria; tx, transporter; str, stroma; x_, external metabolite; GLT, glutamate

(c) The additional essential reactions when photorespiration is involved.

Reaction Name	Reaction	Enzyme	EC No.	LOC
per_RXN969	1.0 per_OXYGEN-MOLECULE + 1.0 per_GLYCOLLATE -> 1.0 per_HYDROGEN-PEROXIDE + 1.0 per_GLYOX	glycolate oxidase	1.1.3.15	LOC_Os03g57220
per_CATRXN	2.0 per_HYDROGEN-PEROXIDE -> 1.0 per_OXYGEN-	catalase	1.11.1.6 ,	LOC_Os02g02400

	MOLECULE			
per_MALATEDEHRXN	1.0 per_MAL + 1.0 per_NAD <> 1.0 per_OXALACETIC_ACID + 1.0 per_NADH	malate dehydrogenase	1.1.1.37	LOC_Os12g43630
per_GLYCINEAMINOTRANSFERASERXN_total	1.0 per_GLT + 1.0 per_SER + 2.0 per_GLYOX -> 1.0 per_OH-PYR + 1.0 per_2-KETOGLUTARATE + 2.0 per_GLY			
per_GLYCERATEDEHYDROGENASERXN	1.0 per_OH-PYR + 1.0 per_NADH -> 1.0 per_GLYCERATE + 1.0 per_NAD	hydroxypyruvate reductase	1.1.1.29/1.1.1.81	
chl_GLY3KINRXN	1.0 GLYCERATE_str + 1.0 ATP_str -> 1.0 ADP_str + 1.0 PGA_str			
chl_GPHRXN	1.0 PGly_str -> 1.0 GLYCOLLATE_str + 1.0 Pi_str			
## transporters##				
per_Mal_tx	1.0 MAL <> 1.0 per_MAL			
per_GLYCERATE_tx	1.0 GLYCERATE <> 1.0 per_GLYCERATE			
per_OAA_tx	1.0 OXALACETIC_ACID <> 1.0			

	per_OXALACETIC_ACID
per_OXYGENMOLECULE_tx	1.0 OXYGEN-MOLECULE <> 1.0 per_OXYGEN-MOLECULE
per_2KETOGLUTARATE_tx	1.0 2-KETOGLUTARATE <> 1.0 per_2-KETOGLUTARATE
Per_GLT_tx	1.0 GLT <> 1.0 per_GLT
chl_GLYCOLLATE_tx	1.0 per_GLYCOLLATE <> 1.0 GLYCOLLATE_str

Per, peroxisome; chl, chloroplast; tx, transporter; str, stroma

Table S3. List of enzymes known to be involved in chlorophyll synthesis.

Enzyme	**EC No.**	**Reference No.**
protoporphyrinogen oxidase	1.3.3.4	3
hydroxymethylbilane synthase	2.5.1.61	3
divinyl chlorophyllide a 8-vinyl-reductase	1.3.1.75	3
chlorophyll synthetase	2.5.1.62	3
2-C-methyl-D-erythritol 2,4-cyclodiphosphate synthase	4.6.1.12	8
uroporphyrinogen decarboxylase	4.1.1.37	3
1-deoxy-D-xylulose-5-phosphate synthase	2.2.1.7	8
protochlorophyllide oxidoreductase	1.3.1.33	35

Glutamine-tRNA ligase/synthetase	6.1.1.17	3
glutamyl-tRNA reductase	1.2.1.70	35;3
glutamate-1-semialdehyde 2,1-aminomutase	5.4.3.8	3
magnesium protoporphyrin IX methyltransferase (ChlM)	2.1.1.11	35
geranyl geranyl reductase (chlP)	1.3.1.83	36
1-deoxy-D-xylulose-5-phosphate reductoisomerase	1.1.1.267	8
4-(cytidine 5'-diphospho)-2-C-methyl-D-erythritol kinase	2.7.1.148	8
magnesium chelatase (chlH)	6.6.1.1	4; 35
coproporphyrinogen III oxidase	1.3.3.3	35; 3
uroporphyrinogen III synthase (UROS)	4.2.1.75	35;3
magnesium protoporphyrin IX monomethyl ester cyclase (CHL27)	1.14.13.81	35
Porphobilinogen synthase/ delta-aminolevulinic acid dehydratase (ALAD)	4.2.1.24	3

Table S4. List of chloroplastic, mitochondrial and peroxisomal transporters added.

Compartment	Transporter	Reaction	Reference No.
Chloroplast	chl_Mal2OG_tx	MAL_str + 2-KETOGLUTARATE <> 2-KETOGLUTARATE_str + MAL	63
	chl_MalGLT_tx	GLT_str + MAL <> MAL_str + GLT	63
	chl_Gln_Glt_tx	GLT + GLN_str <> GLN + GLT_str	68
	chl_NITRATE_tx	NITRATE <> NITRATE_str	
	chl_GLYCOLLATE_tx	per_GLYCOLLATE <> GLYCOLLATE_str	44
	Ammonia_tx	x_ammonia -> Ammonia_str	

	T1	S-ADENOSYLMETHIONINE <> S ADENOSYLMETHIONINE_str	33
	T2	ADENOSYL-HOMO-CYS_str <> ADENOSYL-HOMO-CYS	33
Mitochondria	mit_ammonia_tx	mit_AMMONIA -> AMMONIA_str	59
	mit_SER_tx	SER <> mit_SER	64
	mit_GLY_tx	GLY <> mit_GLY	64
	mit_GLY_o_tx	per_GLY <> mit_GLY_o	44
	mit_SER_o_tx	per_SER <> mit_SER_o	44
Peroxisome	per_OAA_tx	per_OXALACETIC_ACID <> OXALACETIC_ACID	44 for all peroxisomal transporters
	per_GLYCERATE_tx	GLYCERATE_str <> per_GLYCERATE	
	per_Mal_tx	per_MAL <> MAL	
	per_OHPYR_tx	per_OH-PYR -> OH-PYR	
	per_GLT_tx	per_GLT <> GLT	
	per_2KETOGLUTARATE_tx	per_2-KETOGLUTARATE <> 2-KETOGLUTARATE	
	per_LALPHAALANINE_tx	per_L-ALPHA-ALANINE <> L-ALPHA-ALANINE	
	per_PYRUVATE_tx	per_PYRUVATE <> PYRUVATE	
	per_OXYGENMOLECULE_tx	per_OXYGEN-MOLECULE <> OXYGEN-MOLECULE	
	per_GLY_SER_o_tx	1.0 mit_SER_o + 2.0 per_GLY <> 1.0 per_SER + 2.0 mit_GLY_o	

Chl, Chloroplast; mit, Mitochondria; str, Stroma; tx, Transporter; per, Peroxisome; Glu kept as GLT in the model; 2OG kept as 2-KETOGLUTARATE in the model.

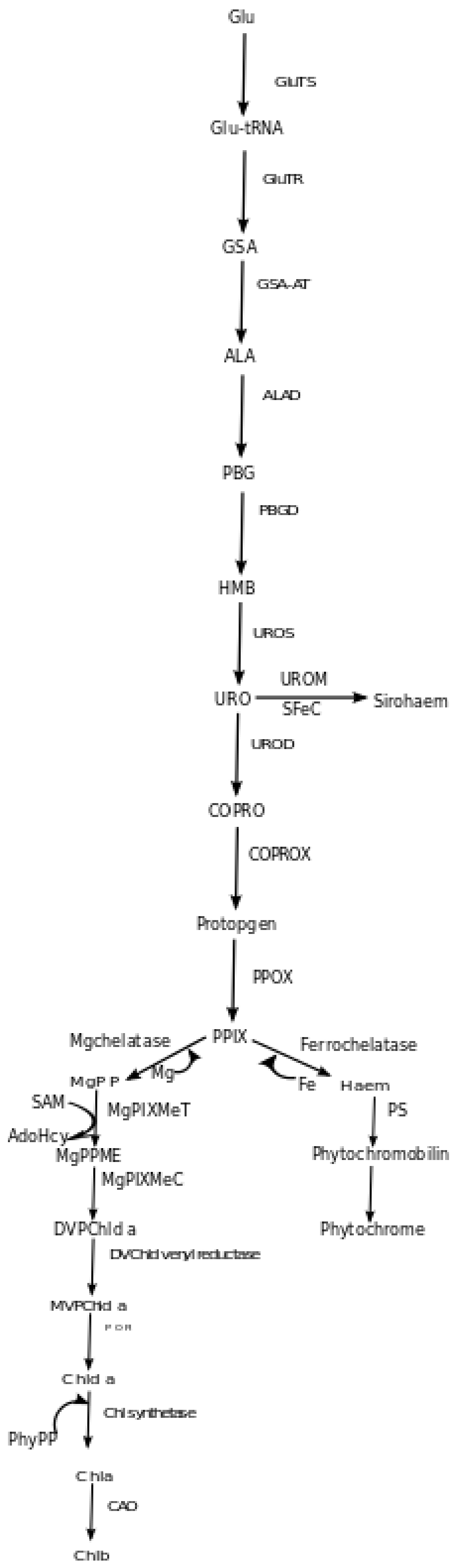

Figure S1. Overview of the chlorophyll biosynthesis pathway and the important steps and the key enzymes involved in it.

Supporting legends

Figure S1. Overview of the chlorophyll biosynthesis pathway and the important steps and the key enzymes involved in it[6]. *Intermediates*: Glu, glutamate; Glu-tRNA, glutamyl tRNA; GSA, glutamate semialdehyde; ALA, 5-aminolevulinic acid; PBG, porphobilinogen; HMB, hydroxymethylbilane; URO, uroporphyrinogen III; COPRO, copro-porphyrinogen III; Protopgen, protoporphyrinogen; PPIX, protoporphyrin IX; MgPP, Mg-protoporphyrin; MgPPME, Mg-protoporphyrinIX monomethyl ester; DVPchld a, divinyl protopchlorophyllide; MVPchld a, monovinyl protochlorophyllide; Chld a, chlorophyllide; Chla, chlorophyll a; Chlb, Chlorophyll b. *Enzymes*: GluTS, Glutamyl-TRNA synthetase; GluTR, Glutamyl-tRNA reductase; GSA AT, Glutamate-1-semialdehyde aminotransferase; ALAD, 5-aminolevulinic acid dehydratase; PBGD, porphobilinogen deaminase; UROS, uroporphyrinogen III synthase; UROM, Urogen III methylase; SFeC,serohaem iron chelatase; UROD, uroporphyrinogen III decarboxylase; COPROX, Coporphyrinogen III oxidase; PPOX, protoporphyrinogenIX oxidase; MgPIXMeT, Mg-protoporphyrin IX methyltransferase; MgPIXMEC, Mg-protoporphyrin IX monomethyl ester cyclase; DV chld Venyl reductase, divinyl chlorophyllide a 8-vinyl-reductase; POR, protochlorophyllide oxidoreductase; Chl synthetase; chlorophyll synthetase; PS, Phytochromobilin synthase; CAO, Chlorophyll a oxygenase.

Supplementary Discussion S1

Elucidating the complete biosynthesis pathway of chlorophyll

We previously mentioned that the existing literature on chlorophyll biosynthesis mainly focused on the pathway starting from Glu. However, from where does this Glu comes from has not been explored. The fact that Glu is the start point for chlorophyll synthesis does indicate participation of many other reactions and compartments for chlorophyll synthesis, but it has not been well documented so far. We tried to decipher the interconnection between the source of Glu and some other intermediates in the chloroplast and the chlorophyll synthesis pathway with the central carbon and nitrogen metabolism. Our results showed that one of the possible chlorophyll biosynthesis pathways has 108 reactions and these are distributed among all the cellular compartments (Fig. 1).

8.0 unit of Glu in the chloroplast is needed for chlorophyll synthesis. Glu in the chloroplast forms ALA via an intermediate glutamate semialdehyde (GSA) which is catalyzed by glutamate-1-semialdehyde 2, 1-aminomutase (GSA-AT). The source of this chloroplastic Glu can be either ferredoxin dependent GOGAT (EC 1.4.7.1) or NADPH dependent GOGAT (EC 1.4.1.13). Our result showed the involvement of the NADPH dependent GOGAT (Fig. 1).

This GOGAT was linked with the chloroplastic Mal-Glu and Mal-2OG transporters. 2-oxoglutarate (2OG) from the cytosol enters into the chloroplast via chloroplastic Mal-2OG transporter and participates in Glu synthesis (EC 1.4.1.13) in the chloroplast. Gln in the chloroplast is produced from ammonia and Glu through glutamine synthase reaction (EC 6.3.1.2). In the chloroplast, one of the important steps of chlorophyll biosynthesis is the conversion of MgPP to MgPPME which is associated with SAM to AdoHcy conversion (Fig. 1). The source of the SAM as substrate within the chloroplast is the chloroplastic SAM-AdoHcy transporter (T1 and T2). The source of Glu in the cytosol is the chloroplastic Mal-Glu transporter. The cytosolic Glu and 3-P-Hydroxypyruvate produces 2OG and 3-Phosphoserine-Serine (3-P-Ser) (EC 2.6.1.52). 3-P-Ser further forms Ser and the reaction is catalyzed by 3-phosphoserine phosphatase (EC 3.1.3.3). The Ser so formed in the cytosol methylates the tetrahydrofolate (THF) and produces Gly and 5,10-methylenetetrahydrofolate (MethyleneTHF) (EC 2.1.2.1). MethyleneTHF consequently produces 5-methyltetrahydrofolate (5-MethylTHF) (EC 1.5.1.20). 5-MethylTHF with homocysteine (Hcy) produces THF and methionine (Met) (EC 2.1.1.14)[32]. Met is the precursor of SAM, which is an essential intermediate in chlorophyll synthesis. Met subsequently produces SAM in the cytosol. This series of reactions that form SAM in the cytosol is called the *methyl cycle*. The Gly so produced is transported to the mitochondrion via mitochondrial Gly transporter. The Hcy required for Met synthesis is produced from AdoHcy which is imported from the chloroplast via a transporter (T2, Fig. 1). Since plant chloroplast and mitochondria lack AdoHcy hydrolase[32], so transport of AdoHcy from the chloroplast to the cytosol is required for methyl cycle to operate[32]. AdoHcy gets converted to Hcy releasing adenosine which with the help of ATP produces AMP and ADP. SAM formed

in the cytosol enters into the chloroplast via SAM transporter (T1, Fig. 1) where it is required for methylation of MgPP to MgPPME, an intermediate for chlorophyll biosynthesis (EC 2.1.1.11) and the produced AdoHcy is to be exported out into the cytosol. It has been reported that impairment of SAM transporter affects plastid biogenesis[33] and moreover lack of SAM synthetase in the chloroplast[32] implies the import of SAM into the chloroplast from the cytosol.

The cytosolic Mal can be transported to the chloroplast via Mal-OAA and Mal-Glu transporter (chloroplastic OAA and Glu are transported to cytosol). On the other hand, chloroplastic Mal can be transported to the cytosol via Mal-2OG transporter. Part of cytosolic OAA is converted to cytosolic citrate and this citrate is transported to the mitochondrion through mitochondrial transporter (Fig. 1).

2OG is also imported from the mitochondrion via 2OG transporter. 2OG is formed through a series of reactions from citrate to isocitrate via cisaconitate (EC 4.2.1.3) and finally to 2OG (EC 1.1.1.42). Gly that enters into the mitochondria combines with THF [catalyzed by glycine decarboxylase, GDC (EC 1.4.4.2)] and gives MethyleneTHF with release of ammonia and carbon-dioxide. Mitochondrial Gly is also involved in the biosynthesis of Ser by serine hydroxymethyltransferase (EC 2.1.2.1) and this Ser comes out of the mitochondrion via mitochondrial Ser transporter. Ser-Gly conversion forms a part of photorespiration as well as folate metabolism. It has been reported that Gly-Ser cycle is obligatory for one carbon metabolism that involves folate and methionine[34]. The mitochondrial ammonia released in GDC mediated reaction is transported into the chloroplast by a transporter where it participates in Gln synthesis from Glu (EC 6.3.1.2). The mitochondrial CO_2 gets released into the cytosol via mitochondrial CO_2 transporter and then gets transported into the chloroplast via chloroplastic CO_2 transporter. CO_2 is also released in three intermediate steps of chlorophyll synthesis (EC 4.1.1.37, 2.2.1.7, and 1.3.3.3). Major part of CO_2 gets involved in Rubisco catalyzed reaction and the remaining gets utilized for synthesis of malate from pyruvate. Light from the external environment (photon) is captured by the chloroplasts and is utilized in light non cyclic (found to be essential) and cyclic (not always) photophosphorylation reactions. Whereas the non-cyclic reaction produces O_2, ATP and NADPH, the cyclic produces only ATP. In the chloroplast, the oxygen gets used up in four intermediate steps of chlorophyll synthesis (1.3.3.4 and 1.14.13.81). However, the major part of O_2 gets exported out of the chloroplast via a transporter and then it gets imported into the mitochondrion to get involved in the electron transport chain.

Clearly, our results indicated how various reactions from different organelles participate in chlorophyll synthesis in plants. More importantly, we observed that besides chloroplast, the other organelles, namely the mitochondria, cytosol and peroxisome (only under photorespiration) are also essentially required to complete the chlorophyll synthesis pathway. Various transporters and few antiporters were found to be required in order to complete the chlorophyll biosynthesis pathway (Fig. 1).
