# Supplementary material for: Revisiting the chlorophyll biosynthesis pathway using genome scale metabolic model of Oryza sativa japonica
Source: Sci Rep. 2015 Oct 7;5:14975. doi: 10.1038/srep14975 (PMC4595741; doi:10.1038/srep14975)
Supplement: Supplementary Data S4 [file srep14975-s5.doc]

Model files:

Chloroplast.xls - reactions in the chloroplast module

chltxs.xls - transporters in the chloroplast module

FromBuild.xls - reactions in the cytosol module

Misc.xls - few more reactions added in the cytosol module

mito.xls - reactions and transporters in the mitochondria module

per.xls - reactions and transporters in the peroxisome module

tx.xls - transporters for biomass components

Abbreviations used in the model files:-

For Compartments/modules:

chl: chloroplast,

mit: mitochondria,

str: stroma;

per: peroxisome,

tx: transporters.
